# Extracorporeal circuit temperature gradient and hemodynamic instability during continuous renal replacement therapy: an observational study

**DOI:** 10.1016/j.aicoj.2026.100108

**Published:** 2026-07-01

**Authors:** Lorna Fraire, Téo Keraron, Matthieu Chivot, Hodane Yonis, Guillaume Chazot, Mehdi Mezidi, Louis Chauvelot, Guillaume Deniel, Jean-Christophe Richard, Laurent Bitker

**Affiliations:** a Service de Médecine Intensive – Réanimation, Hôpital de la Croix Rousse, Hospices Civils de Lyon, France; b Université Claude Bernard Lyon 1, Lyon, France; c Univ Lyon, Université Claude Bernard Lyon 1, INSA-Lyon, CNRS, INSERM, CREATIS UMR 5220, U1294, Villeurbanne, France

**Keywords:** Continuous renal replacement therapy, Temperature management, Hemodynamics, Cardiac output, Mediation

## Abstract

**Introduction:**

Lowering extracorporeal circuit temperature (T°_CRRT_) below core temperature (T°_CORE_) during continuous renal replacement therapy (CRRT) may modify hemodynamic instability (HIRRT) risk by affecting cardiac output (CO) and vasomotor tone. This study aimed to identify the mediators involved in the relationship between CRRT-to-core temperature gradient and HIRRT risk.

**Methods:**

This ancillary analysis of a prospective, single-center center study (NCT03139123) included patients with stage 3 acute kidney injury, receiving CRRT for <24 h and with continuous cardiac index monitoring. T°_CRRT_, T°_CORE_ and hemodynamics parameters were collected 4-hly between inclusion and day 7 (or end of follow-up). Temperature gradient (ΔT°) corresponded to T°_CRRT_ minus T°_CORE_. HIRRT (defined as a mean arterial pressure [MAP] <65 mmHg requiring therapeutic intervention) were reported hourly. An exploratory mediation analysis evaluated the effect of ΔT° on HIRRT risk during follow-up, mediated through CO and MAP.

**Results:**

42 patients were enrolled in this ancillary analysis (age 68 [58–76], SOFA 12 [8–15], 79% with sepsis), and were followed over 119 [57–143] hours (N = 1012 observations). Decreasing ΔT° (extracorporeal cooling) was significantly associated with higher heart rate, CO and MAP, lower norepinephrine dose, and was associated with a reduction in HIRRT risk in univariate analysis (*P* < 0.01). In mediation analysis, ΔT° ≤0°C decreased the probability of HIRRT by 7% (95% confidence interval: 1%–14%, *total effect*). A significant indirect effect was observed (average causal mediation effect [ACME]), operating through MAP and CO mediators (MAP: 5% [2%–7%]; CO: 4% [1%–5%]).

**Conclusions:**

During CRRT, setting the circuit temperature below body temperature was associated with a reduced HIRRT risk, an effect potentially mediated through improvements in MAP and CO.

## Introduction

Hemodynamic instability related to renal replacement therapy (HIRRT) is a frequent complication observed with all renal replacement therapy (RRT) techniques commonly used in the intensive care unit (ICU) [1]. It is associated with higher mortality, uncontrolled fluid balance and poorer renal recovery [2,3]. HIRRT is multifactorial, arising from both the patient's underlying critical illness and inadequate RRT settings that may impair cardiac output (CO) or reduce systemic vosomotor tone beyond the capacity of physiological compensatory mechanisms [1,4].

Continuous RRT (CRRT) is a strong contributor to body heat loss through heat dissipation in the extracorporeal circuit and has led to the use of extracorporeal heating devices to compensate thermal loss [5]. On the other hand, excessive heat transfer to the patient may alter vasomotor tone (arterial but also venous, with an unknown effect on venous return and cardiac preload) and CO, and potentially lead to HIRRT [6]. Cooling the extracorporeal circuit during RRT is an appealing approach to improve hemodynamic stability in critically ill patients. Findings from chronic maintenance hemodialysis show that cooling the dialysate has not uniformly demonstrated a hemodynamic benefit, and underlying mechanisms remain incompletely understood [7,8]. Also, these results cannot be directly extrapolated to the ICU setting, as patients receiving CRRT frequently present with hemodynamic impairment and altered thermoregulation, including frequent hypothermia or hyperthermia.

Evidence specifically addressing CRRT is limited. First, in 21 ICU patients undergoing sustained low-efficiency dialysis, Edrees et al. reported fewer hypotensive episodes with a fixed dialysate temperature of 35 °C, compared to 37 °C [9]. In 30 patients undergoing CRRT, Robert et al. reported that a fixed replacement-fluid temperature of 36 °C was associated with increased mean arterial pressure (MAP) and reduced catecholamine dose over time, although no significant between-group differences were observed compared with a 38 °C setting [10].

These considerations support evaluating CRRT temperature setting relative to the patient's core temperature rather than as a fixed absolute setting, and assessing its hemodynamic effects using advanced hemodynamic monitoring. We hypothesized that setting the extracorporeal circuit temperature (T°_CRRT_) below core temperature (T°_CORE_) during CRRT may be associated with decreased HIRRT risk, through the effects of relative heat loss on vasomotor tone and CO. The primary objective of the study was to evaluate the association between the CRRT-to-core temperature gradient and longitudinal HIRRT risk, and to explore whether this association may be mediated through changes in MAP and CO.

## Methods

### Design

We performed an ancillary analysis of the PRELOAD CRRT study (NCT03139123), a prospective, observational, single-center study conducted between May 2017 and September 2020 in the medical ICU of the Croix Rousse university hospital in Lyon (France), which aimed to describe the prevalence of preload-dependent HIRRT during CRRT [11]. The study was approved by an ethics committee (CPP Ile de France IV, ID-RCB 2017-A00483-50).

### Study population

Eligible patients were adults with Kidney Disease – Improving Global Outcome (KDIGO) stage 3 acute kidney injury, in whom CRRT had been initiated within the 24 h preceding screening for inclusion, and monitored by mean of a calibrated continuous cardiac output (CCO) monitoring device [12]. Exclusion criteria were impossibility or contraindication to perform postural maneuvers (lower limb amputation, inferior vena cava obstruction, pregnancy, intracranial hypertension), decision to withhold treatment, lack of affiliation to social security, legal protective measures, and previous participation to the study.

Patients were followed for a maximum of 7 days from the time of inclusion, or until death, or until the study procedures could no longer be performed (due to discontinuation of CRRT or CO monitoring).

### CRRT and core temperature monitoring

Temperatures were collected 4-hly during the observation period (each 4-hly period corresponding to a visit). T°_CORE_ was continuously monitored at the tip of the PiCCO® arterial catheter (Pulsion Medical, Feldkirch, Germany) positioned in the femoral artery, and automatically reported in electronic ICU charts. 4-hly T°_CORE_ was further categorized into three classes: hypothermia (<36 °C), normothermia (36–37.5 °C), and hyperthermia (>37.5 °C) [13].

T°_CRRT_ corresponded to the temperature set on the CRRT monitor by the clinical team and was collected 4-hly. T°_CRRT_ was adjustable by steps of 1 °C between 35 °C–39 °C on our monitors (Multifiltrate Pro, Fresenius Medical Care, Germany). Effective extracorporeal circuit fluid temperatures were not measured.

### CRRT-to-core temperature gradient definition

Consequently, 4-hly CRRT-to-core temperature gradient (ΔT°, defined as the difference between T°_CRRT_ and T°_CORE_) was negative if T°_CRRT_ was inferior to T°_CORE_, and positive if it was superior to T°_CORE_. 4-hly observations were then classified based on ΔT° into three categories: ≤0 °C resulting in heat loss, between 0–2 °C considered neutral heat transfer, and >2 °C indicating heat gain. These thresholds were empirically defined *a priori*, given the absence of published reference values for the CRRT-to-core temperature gradient in the literature, and considering that a gradient between 0 and 2 °C reflects neutral heat transfer, based on the efficacy of modern integrated heating devices [14].

For descriptive non-analytical purposes, we further classified patients into 3 groups based on the predominant, non-neutral, temperature gradient observed during their total observation period: *predominant heat gain* if more than 30% of the patient’s 4-hly observations had a ΔT° >2 °C; *predominant heat loss* if more than 30% of 4-hly observations had a ΔT° ≤0 °C without fulfilling the above condition; and *predominantly neutral* if none of the 2 preceding conditions were met. The 30% threshold was defined *a priori* on empirical grounds. These groups were not intended for direct comparison, as between-group differences may reflect confounding by indication (as CRRT temperature settings may be driven by patients' clinical status and thermal management requirements).

### CRRT temperature management

Temperature management and T°_CRRT_ setting were not protocolized and left at the discretion of the treating team, using any available means (including external warming blankets) and T°_CRRT_ adjustments. Room temperatures were maintained within a controlled range of 19 °C to 24 °C.

### Participants follow up

The following data were collected prospectively in electronic case report forms every 4 h during follow-up (and matching temperatures collection): MAP, diastolic arterial pressure (DAP), central venous pressure (CVP), heart rate (HR), stroke volume index (SVI), cardiac index (CI), preload dependence status, global end-diastolic volume index, extravascular lung water index, pulmonary vascular permeability index and systemic vascular resistance index. Indexed values were normalized to body surface (Dubois’s formula), and norepinephrine dosage (expressed using the tartrate formulation, corresponding to twice the dose expressed as norepinephrine base) to ICU admission body weight.

### Hemodynamics measurements

We monitored patients with acute circulatory failure with a calibrated continuous cardiac output device using pulse-contour analysis (PiCCO® system, Pulsion Medical, Feldkirch, Germany), with transpulmonary thermodilution calibration performed every 4 h using three injections of normal saline performed on the venous central line. Quality hemodynamic monitoring criteria included correct positioning at the phlebostatic level of all pressure probes (CVP and MAP) and fast flush test verifications to maintain accurate pressure readings. The assessment of preload dependence involved passive leg raising, deemed positive if the CCO increased by more than 10%, or Trendelenburg maneuvers, positive if CCO increased by >8% [15,16].

### HIRRT definition

Episodes of HIRRT, defined as a MAP < 65 mmHg requiring therapeutic intervention (fluid resuscitation, increase or introduction in norepinephrine, UF***_NET_*** decrease or cessation), were also tracked prospectively and reported hourly through end of follow-up. Interventions or events that may have precipitate HIRRT (such as sedation dose or ventilatory settings modifications) were not prospectively collected.

### CRRT settings

The clinician in charge determined the indication, CRRT modality, and settings in accordance with international guidelines and unit protocols [12,17]. CRRT was indicated for patients with acute circulatory failure meeting the initiation criteria of the delayed strategy of the AKIKI trial. CRRT modalities included continuous veno-venous hemofiltration with heparin (CVVH) or continuous veno-venous hemodialysis with regional citrate anticoagulation (CVVHD) using Fresenius Medical systems. CVVH was the preferred technique in all patients, especially in those with an indication for systemic anticoagulation, acute liver failure, or any contraindications to citrate regional anticoagulation (arterial lactate >4 mmol/L, metformin intoxication), or based on clinician’s preference. At the time of the study, regional citrate anticoagulation was reserved for patients with contraindications to systemic anticoagulation. CRRT systems, fluids and settings are described in Supplemental methods 1.

### Statistics

No sample size calculation was performed for the ancillary study; sample size calculation of the PRELOAD CRRT study is reported in the princeps publication. Statistical analyses were preformed using the R software (version 4.1.3) [18,19]. A *P* value under 0.05 was used to define statistical significance. Continuous data are reported using median [interquartile range] and categorical data using count (percentage).

The study’s analytical unit was the 4-hly observation. The analysis was performed on all included patients, including those whose observation period did not reach day 7. Missing variables during study participation (inclusion to day 7, or end of follow-up, whichever occurred first) were imputed on 10 datasets by predictive mean matching for continuous variables and logistic regression for categorical variables [20]. End of follow-up corresponded to any of the following event occurring during the 7-day observation period: death, CRRT weaning, or end of calibrated CO monitoring.

In all following models and regression analyses described below, mixed effects were used with the visit number (continuous) as the random slope nested in a random intercept corresponding to the patient identification number [19]. Variables were scaled and centered prior to regression; transformation was considered in case of significant skewness.

Because temperature gradient was correlated with T°_CORE_ (coefficient of correlation *r* 0.73, coefficient of determination *R^2^* 0.53) with a subsequent substantial risk of confounding by indication, while being also potentially affected by other covariates (age, sepsis, invasive mechanical ventilation, CRRT set blood flow and sedation), an inverse probability of treatment weighting (IPTW) vector was determined using the covariate balancing propensity score (CBPS, Supplemental Fig. S1) to weight models’ coefficients [21]. Sensitivity analyses were performed without CBPS weighting.

Association of temperature gradient with 4-hly hemodynamics measured during the same visit was first performed using categorized ΔT° within each T°_CORE_ categories in bivariate analysis (including the evaluation of the interaction between the 2 explanatory variables). Then, the association of ΔT° with hemodynamics was evaluated using ΔT° as a continuous explanatory variable, with or without CBPS weighting. A sensitivity, unweighted, analysis was performed in the subset of observations with a normal T°_CORE_ (between 36 °C and 37.5 °C) to account for the potential bias associated with hypo- or hyperthermic observations.

Univariate association of demographics, severity of disease variables, longitudinal hemodynamics and weighted temperature gradient with short-term HIRRT risk (i.e. the risk of presenting an HIRRT in the following 4 h of the index visit) during follow-up were evaluated using generalized linear mixed effects regression models (using the binomial law), expressed using their odds ratio and corresponding 95% confidence interval. A sensitivity analysis of HIRRT risk prediction by temperature gradient in observations with a normal T°_CORE_ was also performed, without CBPS weighting (as it principally corrected for the effect of core temperature). This was followed by a multivariate regression analysis, using variables deemed physiologically relevant to predict HIRRT risk [5]. Multicollinearity and interactions were systematically checked for. Quadratic factors applied to continuous predictors were also tested. The models’ calibration and goodness-of-fit were evaluated using the C-statistic, calibration plots (in weighted models) and the Kolmogorov–Smirnov test (DHARMa R package, in unweighted models), respectively [22].

### Exploratory mediation analysis

Supplemental Fig. S2 shows the hypothesized causal relation existing between ΔT° (treatment) and HIRRT risk (outcome), mediated through MAP and CO (mediators) by mean of directed acyclic graph. MAP and CO were considered as principal mediators, as they represent the hemodynamic determinants of tissue perfusion and direct physiological links between extracorporeal heat transfer and hemodynamic instability. Because MAP was one of the criteria defining HIRRT (in conjunction with predefined therapeutic interventions), a sensitivity analysis was also considered using diastolic arterial pressure (DAP), instead of MAP, given its recognized relation with systemic vascular resistances [23]. Details regarding causal mediation analysis methodology are given in Supplemental methods 2.

## Results

### Cohort description

Forty-two patients were enrolled and were followed over 119 [57–143] hours, for a total of 1012 4-hly observations. Median age was 68 [58–76], 62% patients were males, the Sepsis-related Organ Failure Assessment score was 12 [8–15], and 52% were in septic shock at time of inclusion. Patients’ characteristics at inclusion are reported in Table 1. The delay between ICU admission and CRRT initiation was 1 [0–3] days. The CRRT settings at inclusion were similar between the 3 groups and the predominant CRRT modality was CVVH. Baseline hemodynamics are shown in Supplemental Table S2.

**Table 1:** Patient baseline characteristics, CRRT settings and clinical outcomes.

Variables	All patients	Predominant heat gain (ΔT° > 2 °C)	Predominant neutral (ΔT° 0°–2 °C)	Predominant heat loss (ΔT° ≤ 0 °C)
	N = 42	N = 16	N = 12	N = 14
Age, years	68 [58–76]	70 [64–82]	73 [61–78]	58 [49–68]
Sex (male), N (%)	26 (62%)	11 (69%)	7 (58%)	8 (57%)
Body mass index, kg.m^−2^	26 [22–31]	25 [21–28]	27 [24–32]	26 [21–30]
Body weight at inclusion, kg	74 [69–86]	74 [66–80]	75 [71–84]	72 [68–89]
SAPS-2 score	64 [49–76]	66 [60–81]	52 [46–72]	68 [56–76]
SOFA score	12 [8–15]	12 [8–14]	10 [8–12]	14 [11–16]
Non-cardiovascular SOFA score	8 [5–11]	8 [4–11]	6 [5–8]	10 [7–12]
Non-renal SOFA score	9 [6–12]	9 [3–11]	7 [6–8]	12 [9–12]
Medical admission context, N (%)	42 (100%)	16 (100%)	12 (100%)	14 (100%)
*Comorbidities*				
Diabetes, N (%)	12 (29%)	4 (25%)	4 (33%)	4 (29%)
Chronic respiratory disease, N (%)	4 (10%)	0 (0%)	3 (25%)	1 (7%)
Chronic heart failure, N (%)	10 (24%)	3 (19%)	5 (42%)	2 (14%)
Coronary artery disease, N (%)	12 (29%)	4 (25%)	4 (33%)	4 (29%)
Cirrhosis, N (%)	6 (14%)	4 (25%)	2 (17%)	0 (0%)
*Acute circulatory failure cause*				
Cardiogenic shock, N (%)	8 (19%)	1 (6%)	5 (42%)	2 (14%)
Septic shock, N (%)	22 (52%)	9 (56%)	3 (25%)	10 (71%)
Vasoplegic non-septic shock, N (%)	8 (19%)	3 (19%)	4 (33%)	1 (7%)
Post-cardiac arrest syndrome, N (%)	4 (10%)	3 (19%)	0 (0%)	1 (7%)
Sepsis, N (%)	33 (79%)	14 (88%)	6 (50%)	13 (93%)
Septic shock, N (%)	24 (57%)	11 (69%)	3 (25%)	10 (71%)
Invasive mechanical ventilation, N (%)	35 (83%)	13 (81%)	8 (67%)	14 (100%)
RASS score, N (%)	−5 [−5–−4]	−5 [−5–−1]	−5 [−5–0]	−5 [−5–−5]
Neuromuscular blockade agonist, N (%)	18 (43%)	4 (25%)	4 (33%)	10 (71%)
Delay between ICU admission and CRRT start, h	1 [0–3]	1 [0–3]	2 [0–3]	1 [0–4]
Delay between CRRT start and inclusion, h	6 [1–15]	8 [3–14]	5 [1–16]	4 [1–17]
Fluid balance at inclusion, kg	3 [0–8]	4 [0–8]	2 [−3–4]	4 [1–10]
*CRRT technique*				
CVVH, N (%)	39 (93%)	14 (88%)	12 (100%)	13 (93%)
CVVHD, N (%)	3 (7%)	2 (12%)	0 (0%)	1 (7%)
*CRRT anticoagulation technique*				
Regional citrate anticoagulation, N (%)	3 (7%)	2 (12%)	0 (0%)	1 (7%)
Intravenous heparin, N (%)	39 (93%)	14 (88%)	12 (100%)	13 (93%)
*CRRT settings*				
Blood flow, ml.min^−1^	250 [200–250]	250 [200–250]	250 [250–250]	250 [200–288]
Effluent flow rate, ml.kg^−1^.h^−1^	29 [26–32]	29 [24–32]	29 [26–31]	29 [27–34]
Net ultrafiltration flow rate, ml.h^−1^	0 [0–200]	44 [0–300]	50 [0–162]	0 [0–188]
Net ultrafiltration flow rate, ml.h^−1^.kg^−1^	0 [0–2.9]	0.7 [0–4.5]	0.6 [0–2.9]	0 [0–2.7]
*Clinical outcomes*				
Death at day-90, N (%)	26 (62%)	13 (81%)	8 (67%)	5 (36%)
RRT dependence at day-90, N (%)	0 (0%)	0 (0%)	0 (0%)	0 (0%)

Data is median [interquartile range] or count (percentage).

Longitudinal groups of predominant temperature gradient categories (columns 2–4) were defined as fraction of time spent >2 °C or ≤0 °C greater than 30% of total follow-up. Groups were defined solely for descriptive purposes based on the predominant temperature gradient observed during follow-up and are not intended for direct comparison.

CVVHD: continuous veno-venous hemodialysis; CVVH: continuous veno-venous hemofiltration; CRRT: continuous renal replacement therapy; ΔT°: temperature gradient; ICU: intensive care unit; RASS: Richmond analgesia and sedation scale; RRT: renal replacement therapy; SAPS-2: simplified acute physiology score 2; SOFA: sequential organ failure assessment.

### Temperature course over time

Supplemental Table S1 shows the core temperature values and gradients at baseline in the cohort. Most patients were in the normothermia T°_CORE_ group (36–37.5 °C). Of note, only one patient had a core temperature >38.3 °C at inclusion.

**Fig. 1:**
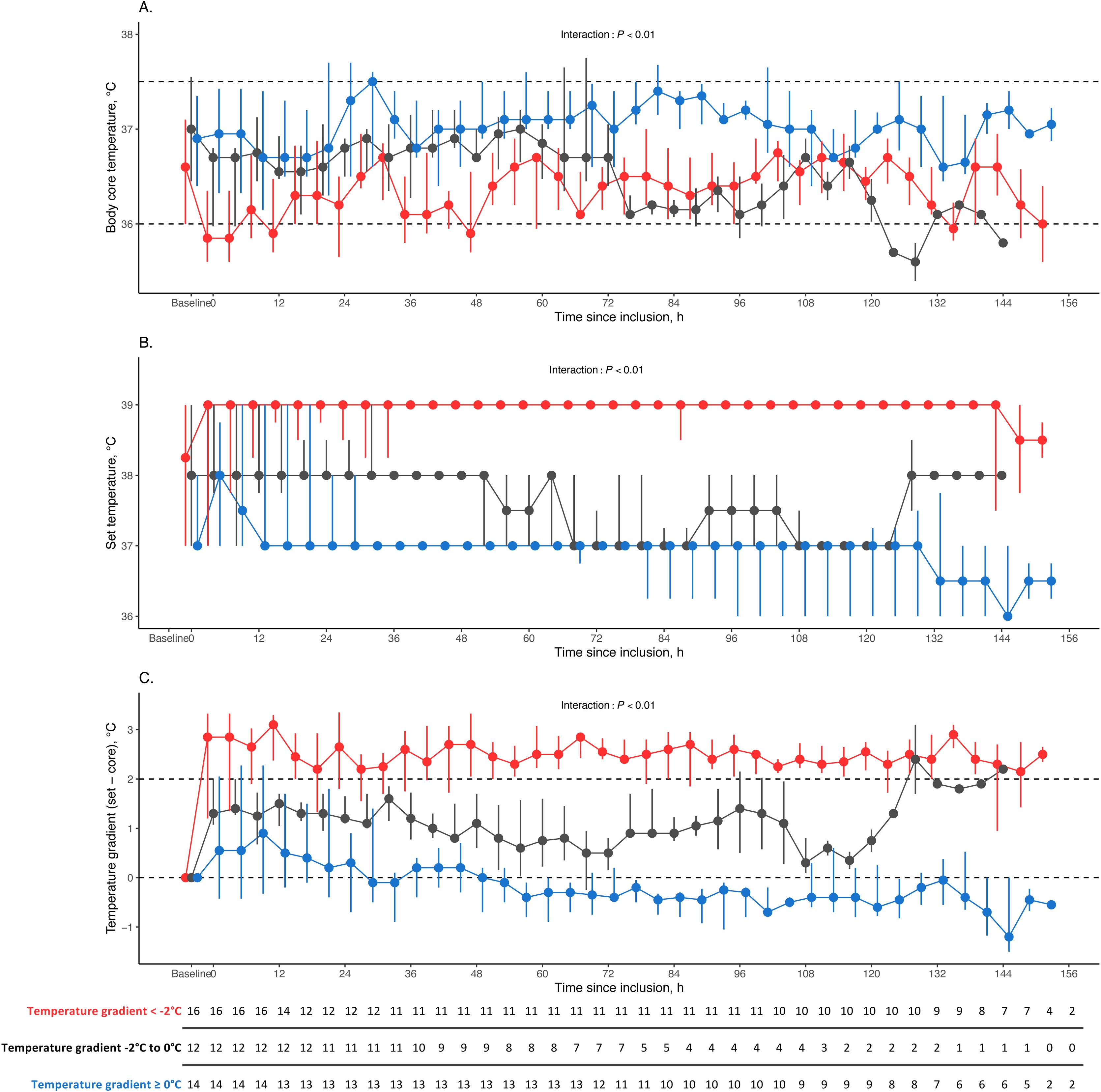
Core temperature, CRRT set temperature and temperature gradient over time. The figure shows the median value and first and third quartile value of core temperature (A), CRRT set temperature (B) and temperature gradient (C) over time in the cohort, categorized by the fraction of time spent in a temperature gradient category (>30% of time with temperature gradient ≤0 °C in blue, >30% of time with temperature gradient >2 °C in red, the remaining observations in grey). Below the panels are the number of patients at risk in each category over time. In each panel, the *P* value examines the association of the interaction existing between elapsed time since inclusion (categorical) and the gradient category with the variable of interest, using a linear mixed effects model, with the patient identification number as the random intercept and the elapsed time as a random effect (continuous). An offset for the core temperature measured before inclusion was also included. CRRT: continuous renal replacement therapy.

Median ΔT° during follow-up was 1.3 [−0.1–2.4] °C (Fig. 1, and Supplemental Fig. S3 for the individual temperature course over time). Supplemental Figure S4 shows the relationship between T°_CORE_, T°_CRRT_, and temperature gradient, based on the predominant ΔT° group. Of note, no negative gradient was observed when core temperature was below 36 °C, and gradients >2 °C were not observed when core temperature was >37.5 °C (Supplemental Figure S5).

### Association of 4-hly temperature gradient with hemodynamics

**Fig. 2:**
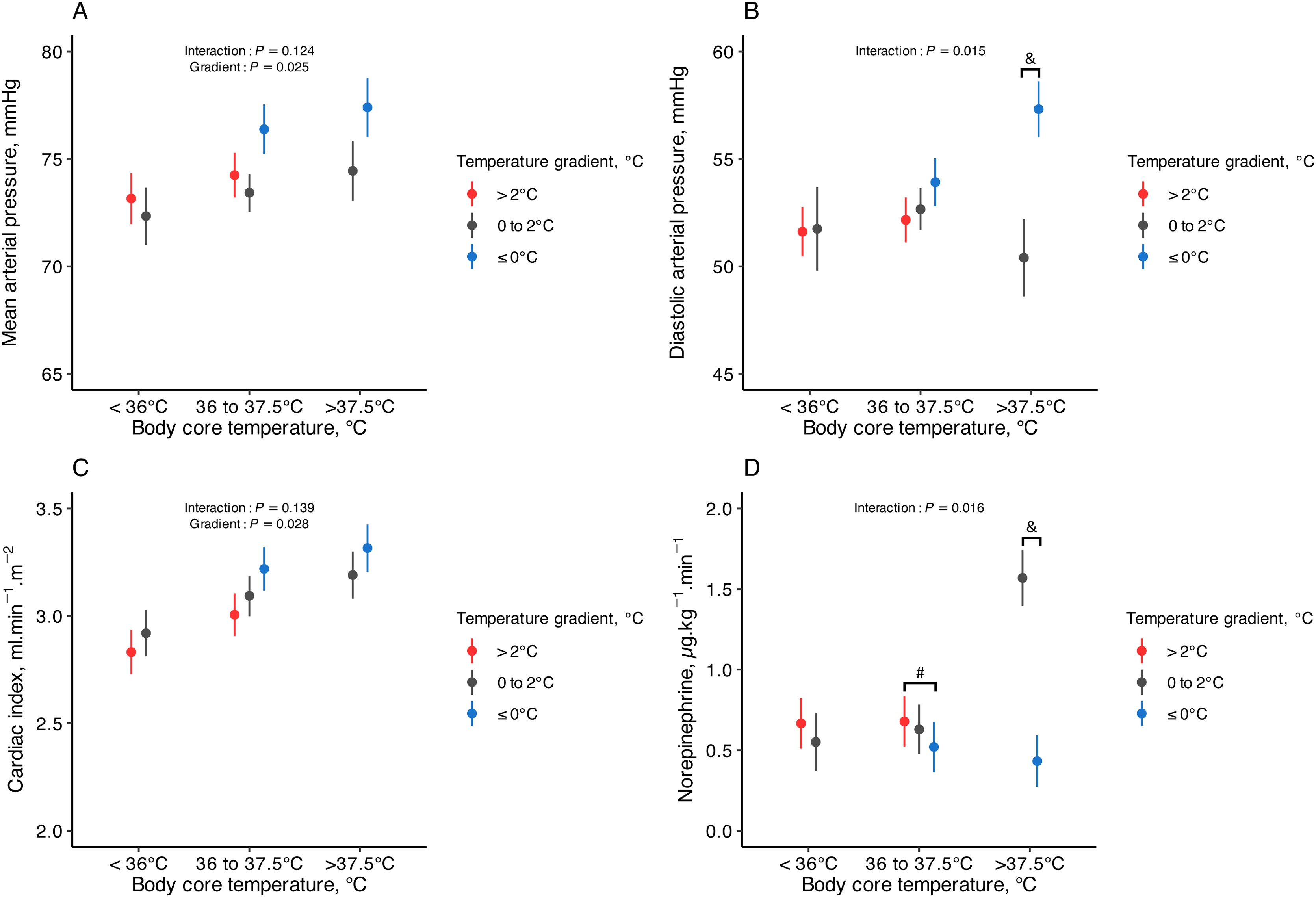
Association of hemodynamic parameters with temperature gradient and core temperature. The figure shows the mean value of mean arterial pressure (A), diastolic arterial pressure (B), cardiac index (C), and norepinephrine dose (D) based on the core temperature category (x axis) and the temperature gradient (>2 °C in red, between 0 and 2 °C in grey, and ≤0 °C in blue) during longitudinal follow-up (4-hly observations). The represented values are the marginal means (and associated standard error) determined from a mixed effect model with the hemodynamic parameter as the dependent variable, temperature gradient category and core temperature category as the explanatory variables (with an interaction term if significant). Models’ random effects were a random slope of visit number nested in a random intercept corresponding to the patient identification number. An offset was inserted in the model, corresponding to the hemodynamic parameter value at baseline. Marginal means were determined in 10 imputed datasets (N = 1012 observations in each dataset), and pooled using Rubin’s method. Variables were scaled prior to regression (with additional Box-Cox transformation for norepinephrine due to leftward skewness) and descaled after. For all variables, interaction between the 2 explanatory variables was checked. If significant, a post-hoc pairwise comparison was performed using Sidak’s method. P values were bootstrapped over 500 replicate datasets. Collinearity between the 2 categorical variables was eliminated using a variance inflation factor <3. #: *P*<0.05 between gradient category >2 °C (red) and ≤0 °C (blue) in post-hoc pairwise analysis; &: *P* < 0.05 between gradient category 0 °C–2 °C (grey) and ≤0°C (blue) in post-hoc pairwise analysis.

A ΔT° ≤0 °C in normothermic and hyperthermic T°_CORE_ observations was associated with significantly higher MAP and DAP, CO, heart rate and lower norepinephrine dose (Fig. 2 and Supplemental Figure S6, Supplemental Figure S7 for observed values). When considered continuously, a 1 °C decrease in 4-hly ΔT° (heat loss) was associated with a small yet significant increase in MAP, DAP, heart rate and CO (Table 2).

**Table 2:** Association of temperature gradient with hemodynamic parameters during follow-up.

	Temperature gradient, per 1 °C decrease					
Variables (4-hly observations)	Unweighted All observations	*P* value	Weighted All observations	*P* value	Unweighted Normothermic observations*	*P* value
Mean arterial pressure, mmHg	1.1 ± 0.3	<0.01	1.1 ± 0.4	0.01	1.1 ± 0.4	0.02
Diastolic arterial pressure, mmHg	0.9 ± 0.3	<0.01	0.9 ± 0.3	0.01	0.7 ± 0.3	0.02
Cardiac index, L.min^−1^. m^−2^	0.10 ± 0.02	<0.01	0.07 ± 0.02	<0.01	0.09 ± 0.03	<0.01
Heart rate, min^−1^	2.9 ± 0.4	<0.01	2.2 ± 0.5	<0.01	2.5 ± 0.6	<0.01
Stroke volume index, ml.m^−2^	−0.1 ± 0.2	0.61	−0.1 ± 0.3	0.55	−0.1 ± 0.3	0.76
Relative change in CCI during postural maneuver, %	0.5 ± 0.3	0.15	0.2 ± 0.4	0.57	0.7 ± 0.5	0.33
Norepinephrine dose (tartrate), μg.kg^−1^.min^−1^	−0.04 ± 0.02	0.13	−0.08 ± 0.02	0.01	−0.04 ± 0.02	<0.01

Data is estimate ± standard error.

*: observations with a core temperature between 36 °C and 37.5 °C (N = 753).

Mixed effects linear regression models were run on 10 imputed datasets (N = 1012 observations in each dataset), with temperature gradient as the fixed effect, the hemodynamic variable as the dependent variable, visit number as the random slope nested in a random intercept corresponding to the patient identification number. Variables were scaled and centered prior to regression (norepinephrine required additional transformation using the Box-Cox method due to leftward skewness). Fixed effects were then pooled using Rubin’s rule and descaled to the original hemodynamic parameter scale. Models included an offset corresponding to the baseline hemodynamic value at time of inclusion. Weighting was performed using the CBPS method to adjust for the effect of pre-treatment confounders on temperature gradient. P values were bootstrapped over 500 replicate datasets.

CCI: continuous cardiac index by pulse contour analysis.

### HIRRT risk and 4-hly temperature gradient

The overall number of HIRRT episodes was 214, with a median number of episodes per patient of 4 [3–8] during follow-up (Supplemental Table S1). Supplemental Figure S8 shows the rate of HIRRT episodes based on 4-hly ΔT° category in each predominant ΔT° group. Interventions associated with HIRRT episodes did not significantly differ based on the 4-hly ΔT° category, nor did it seem to be significantly associated with a subsequent change in CRRT temperature setting (Supplemental Figure S9).

**Fig. 3:**
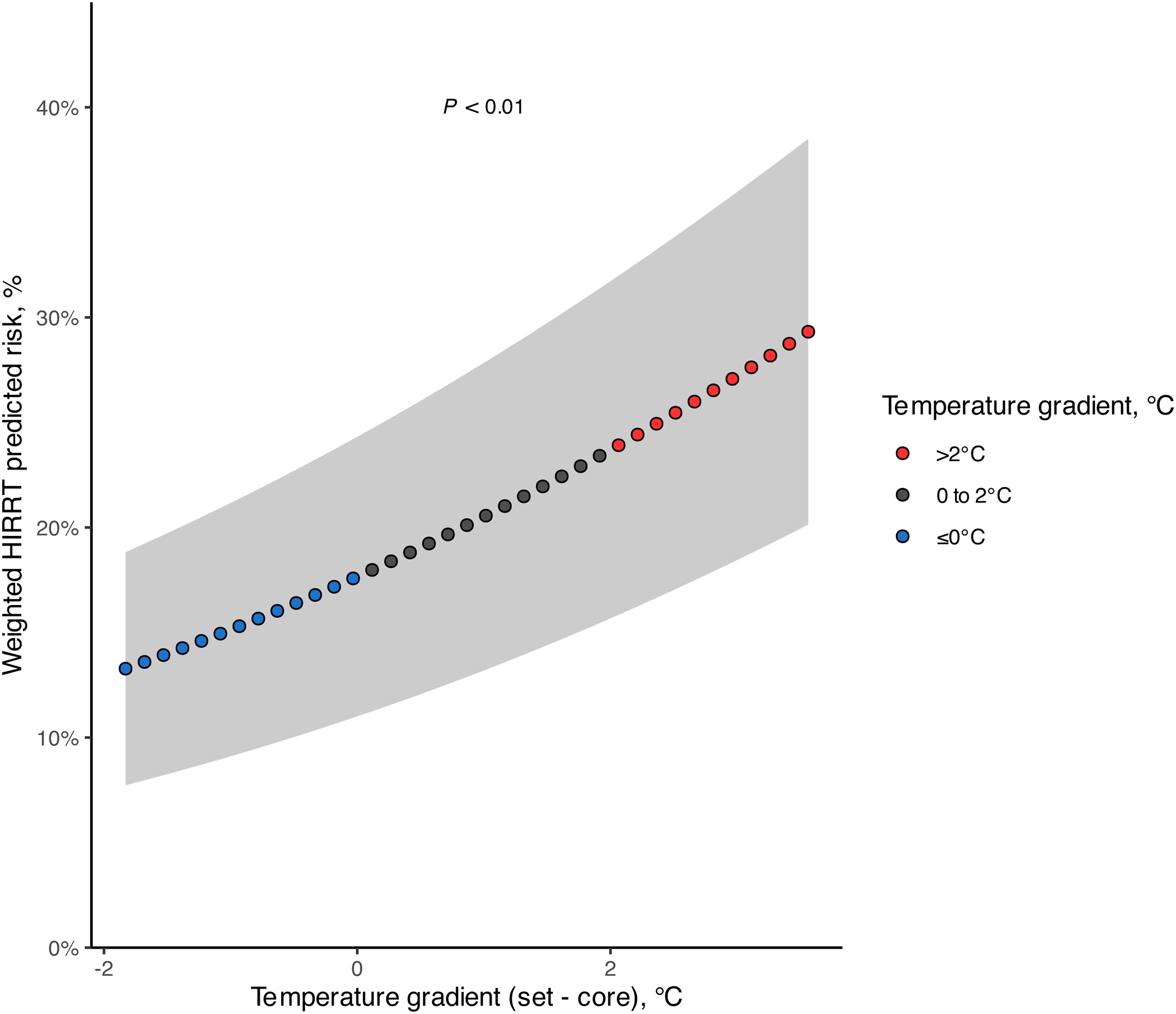
Association of temperature gradient with longitudinal HIRRT risk during follow-up (CBPS-weighted analysis). The figure shows the predicted risk of HIRRT associated with temperature gradient during follow-up (*P* < 0.01). Gradient categories are also represented. The grey shade represents the standard deviation of the prediction. Prediction was performed using a generalized linear regression mixed effects model, with HIRRT as the dependent variable, temperature gradient as the explanatory variable, and applied to 10 imputed datasets. Models’ random effects were a random slope of visit number (longitudinal follow-up) nested in a random intercept corresponding to the patient identification number. Model was weighted for the core temperature, using the CBPS method. Model coefficients were then pooled, and applied to a synthetic dataset with temperature gradient varying between the lowest and highest value observed in the cohort. For each imputed datasets, posterior predictive checks were performed using calibration plots and the C-statistic (and its 95% confidence interval, Delong’s method) was computed, and then pooled (final model’s C-statistics: 0.59 [95% confidence interval: 0.57–0.61]). CBPS: covariate balancing propensity score; HIRRT: hemodynamic instability associated with renal replacement therapy

In CBPS-weighted univariate analysis, higher ΔT° was associated with a significant increase in HIRRT risk (Fig. 3), which was also confirmed in the subset of normothermic observations (Supplemental Figure S10). In multivariate analysis (which included co-adjustment with the potential hemodynamic mediators), ΔT° was not significantly associated with HIRRT longitudinal risk (Table 3, univariate analyses presented in Supplemental Table S2). Of note, no significant interaction existed between MAP and CO with HIRRT risk prediction.

**Table 3:** Multivariate analysis of variables associated with longitudinal HIRRT risk.

	HIRRT risk in the following 4 h	
Variables (4-hly observations)	Odd ratio [95% c.i.]	P value
Mean arterial pressure, per 10 mmHg increase*		<0.01
First degree component	0.08 [0.02–0.38]	
Second degree component	1.01 [1.00–1.02]	
Cardiac index, per 0.1 L.min^−1^.m^−2^ increase	0.99 [0.96–1.02]	^$$^
Relative change in CCI during postural maneuver, per 1% increase	1.15 [1.06–1.24]	^$$^
Relative change in CCI × cardiac index (*interaction term*)	1.00 [0.99–1.00]	^$$^
Norepinephrine dose (tartrate), per 0.1 μg.kg^−1^.min^−1^ increase	-^||^	–
Temperature gradient, per 0.1 °C increase (unweighted)	1.01 [0.99–1.02]	0.33
Non-cardiovascular SOFA score, per 1 point increase	0.94 [0.90–0.98]	<0.01
Delay since last HIRRT <8 h (reference is ≥8 h)	-^||^	–
Daily lactate, per 1 mmol. L^−1^ increase	2.40 [1.23–4.69]	0.02

*: quadratic polynomial factor in the model.

^||^: not retained in the model after backward stepwise selection.

^$$^: significant interaction term between cardiac index and relative change in CCI (*P*<0.05).

The mixed effects generalized linear regression model were run on 10 imputed datasets (N = 970 observations in each dataset, due to the absence of follow-up in the next 4 h regarding HIRRT at that time point), with the variables of interest as the fixed effects, HIRRT as the dependent variable, visit number as the random slope nested in a random intercept corresponding to the patient identification number. Variables inserted in the initial model were those with a P value < 0.20 in univariate analysis and deemed physiologically relevant to predict HIRRT risk. Variables were scaled and centered prior to regression (norepinephrine required additional transformation using the Box-Cox method due to leftward skewness). Backwards stepwise model selection was performed using Akaike’s information criterion. Variables retained in the final model were those retained in at least 8 of the 10 imputed datasets (delay since last HIRRT <8 h: 4 votes, norepinephrine dose: 4 votes). Fixed effects were then pooled using Rubin’s rule and descaled to the original hemodynamic parameter scale. No CBPS weighting was applied in this analysis. For each imputed datasets, the C-statistics (and its 95% confidence interval, Delong’s method) and their results pooled, and the models’ goodness-of-fit were checked using Hartig et al. method (R package *DHARMa,* Kolmogorov–Smirnov goodness-of-fit test). Final model’s C-statistics: 0.78 (95% confidence interval: 0.75–0.82), Kolmogorov–Smirnov test: *P* = 0.52.

95% c.i.: 95% confidence interval; CCI: continuous cardiac index by pulse contour analysis; HIRRT: hemodynamic instability related to renal replacement therapy; SOFA = sepsis-related organ failure assessment.

### Exploratory mediation analysis

In mediation analyses (Supplemental Figure S11), a ΔT° ≤0 °C was significantly associated with a decrease in HIRRT risk mediated through both mediators (MAP and CO, indirect effect), while the direct (unmediated) effect of ΔT° on HIRRT risk was non-significant, suggesting full mediation. The total (direct and indirect) effect of ΔT° on HIRRT risk was also significant, with a risk reduction of 7% (95% confidence interval: 1%–13%). ΔT° did not appear to significantly alter the mediator’s effects on HIRRT risk. Sensitivity analyses varying the ρ parameter suggested that the mediation results were robust to small-to-moderate violations of the sequential ignorability assumption, but sensitive to larger unmeasured confounding. Supplemental Figures S12 and S13 show results of the prespecified sensitivity mediation analyses (normothermic observations, and using DAP instead of MAP).

## Discussion

In this prospective, observational, single center, mediation study of a cohort of patients treated with CRRT and receiving advanced hemodynamic monitoring, we found that a decreasing ΔT° (i.e. body heat loss) was significantly associated with higher CO and MAP, as well as with a lower HIRRT risk. A negative temperature gradient (ΔT° ≤0 °C, i.e. a CRRT temperature setting equal or inferior to core temperature) was significantly associated with a reduction in HIRRT risk, compared to a ΔT° >0 °C, an effect than was statistically mediated by its effects on MAP and CI.

A cool dialysate strategy (usually 1 or 2 °C below body temperature) is usually considered effective in patients undergoing maintenance hemodialysis on preventing intradialytic hypotension, although conclusions of a recent cluster randomized controlled trial (using a temperature gradient between 0.5 to 0.9 °C below body temperature) do not support this practice [8,[24], [25], [26]]. In the context of critical illness, Schortgen and al. demonstrated that cooling the dialysate to lower body temperature, along with other interventions, reduced the risk of intradialytic hypotension in critically ill patients undergoing intermittent hemodialysis [27]. Edrees and al. also observed in a prospective randomized cross-over pilot study of patients receiving sustained low-efficiency dialysis (SLED) that a cool dialysate strategy led to significantly less HIRRT compared with dialysate temperature of 37 °C [9]. Finally, during CRRT, Robert and al. reported that setting the replacement fluids’ temperature to 36 °C had no impact on body temperature but led to increased MAP and decreased catecholamine infusion dosage (in line with our results), but were unable to conclude regarding HIRRT risk prevention given its small sample size [10].

We observed that lower temperature gradients were associated with higher heart rate and CO, and this effect may have translated into a protective effect on HIRRT risk in mediation analysis. External cooling (applied to the peripheral/cutaneous envelope) is known to generate hyperdynamic states, with an increase in CO, energy expenditure and oxygen consumption (VO_2_) in exercising healthy subjects, in relation with the increase heart rate, muscle mass activity (shivering) and hypothalamus-driven neurohormonal (adrenergic) activation aiming to maintain core temperature constant [28]. These effects are associated with regional metabolic vasodilation in thermogenic tissues which offsets cutaneous vasoconstriction, leading to maintained or reduced total peripheral vascular resistance [29]. Oppositely, intensive therapeutic cooling of septic and febrile (sedated) patients with invasive mechanical ventilation has repeatedly showed a marked decrease in CO, VO_2_ and energy expenditure, especially in sedated, paralyzed patients [27,30,31]. Among those, Rokyta et al. observed that cooling patients undergoing CRRT (using a replacement fluid temperature of 20 °C), with septic shock and hyperthermia (>37.5 °C) led to decreased VO_2_, CO and heart rate [6]. These contrasting results are related to the fact that these studies applied intensive cooling to generate a drastic fall in body core temperature (by cooling the dialysate down to 20 °C or by turning off the heater, implying a ΔT° of −17 °C or less), when our population was exposed to an absolute difference between CRRT temperature and core temperature of ±2 °C.

The CRRT-to-core temperature gradient is mathematically dependent on core temperature, such that a negative gradient may reflect a deliberate clinical decision to attenuate hyperthermia or limit heat gain, especially in the absence of a protocolized temperature management strategy. This is supported by the observation that all negative gradient observations occurred in patients with a core temperature above 36 °C, with the majority falling within the normal core temperature range. As a result, the three descriptive patient groups defined by predominant ΔT° were markedly heterogeneous, reflecting potential confounding by indication. Differences in clinical severity and hemodynamics between groups (e.g. higher heart rate and vasopressor dose in the predominant ΔT° ≤0 °C group, or a mortality rate >80% in the predominant ΔT° >2 °C group) likely reflect differences in underlying core temperature and illness severity as much as any effect of extracorporeal heat transfer itself. This interdependence provided strong rationale for performing the prespecified sensitivity analysis of the longitudinal data in the subgroup of normothermic observations, and the use of IPTW using CBPS adjusting for the confounding effect of core temperature.

Unlike therapeutic or inadvertent hypothermia (which imply an absolute reduction in core temperature), a ΔT° ≤0 °C reflects a relative heat loss from the extracorporeal circuit, which may attenuate heat gain or slow the rise in core temperature without necessarily inducing even mild hypothermia. In that sense, Robert et al. observed that core temperatures did not significantly vary over time with the application of either lower or higher extracorporeal temperature strategies (36 °C or 38 °C), with no patients experiencing core temperatures <35.5 °C [10]. Profound hypothermia is a known contributor of coagulopathy in trauma patient, and cooling patients undergoing CRRT could lead to similar complications by inhibiting the initiation phase of thrombin generation and fibrinogen synthesis [32]. Indeed, a critical body temperature of 34 °C has been identified, below which platelet function is significantly impaired, a temperature that was not observed in our cohort [33]. Also, exposure to cold temperature affects cellular and molecular defenses against pathogens in both humans and animals and causes secretion of norepinephrine and cortisol, decreased lymphoproliferative responses, suppression of the innate immune function and alteration of cytokine production, with an existing association with increased septic complications and mortality [34]. Also, mild to more intense extracorporeal cooling may lead to shivering and increased muscle metabolic demand in non-paralyzed patients, hence aggravating regional tissue hypoxia. Finally, the incidence of cold sensations in awake patients should also be accounted for [35,36]. These important elements were not, however, evaluated in our study.

Causal mediation analysis relies on the sequential ignorability assumption, which requires the absence of unmeasured confounding in both the exposure–mediator and mediator–outcome relationships. Sensitivity analyses suggested that the mediation results were robust to small-to-moderate violations of this assumption but sensitive to larger degrees of unmeasured confounding. This is expected in a complex critical care setting where multiple simultaneous interventions (e.g. sedation dose or ventilatory settings modifications, antipyretic agents administration) and unmeasured physiological interactions cannot be fully accounted for. Among these expected biases is the fact that CRRT temperature was left at the discretion of the treating team, and may not have been completely accounted for by IPTW-CBPS adjustment. Although longitudinal causal frameworks such as G-estimation or targeted maximum likelihood estimation (TMLE) would theoretically be more appropriate to handle time-varying confounding and treatment–mediator feedback, their application was not feasible given the size of our cohort. These analyses should therefore be interpreted as exploratory and hypothesis-generating, and interventional studies are needed to confirm these findings.

The principal strength of our study is the highly documented longitudinal hemodynamic monitoring (including CO assessment) of a cohort of patients with multi-organ failure. The use of advanced hemodynamic monitoring in patients undergoing CRRT has gained increasing interest, as it may inform clinicians on the underlying mechanisms of HIRRT or help guide fluid removal strategies [1,37,38]. Second, this is the first description of the impact of the circuit-to-core temperature gradient in the CRRT setting, a temperature management strategy which accounts for variations in the patient’s body core temperature.

Yet, the study has also several limitations that must be addressed. First, because of the single-center study design, extrapolation of our results to other ICUs may be questionable. Second, our definition of HIRRT was limited to hypotensive episodes requiring therapeutic interventions and did not account for other presentations of hemodynamic impairment (e.g. mottling, tachycardia). Furthermore, differences in practice may have impacted event classification, given the absence of therapeutic interventions’ protocolization. Also, MAP as a mediator may generate circularity, being a component of the used HIRRT definition. However, the sensitivity analysis using DAP instead of MAP demonstrated similar results. Third, we did not report skin temperature (a potent mediator of core temperature control) and effective CRRT circuit temperature, which limits the physiological interpretation of the tested conditions. Likewise, the effective temperature of the venous blood returning to the patient was not measured, and would have more precisely quantified the heat transfer occurring in the extracorporeal circuit [14]. Furthermore, other CRRT platforms apply external heating not on the substitution/dialysate fluids, but on the blood lines, which may limit the generalizability of our results. Consequently, the empirical threshold defining ΔT° categories may not hold if other systems are used. However, no prior evidence supported the use of alternative thresholds, and the analysis of ΔT° as a continuous variable suggests that a single threshold may be an oversimplification, despite its clinical practicality. Fourth, although advanced hemodynamic monitoring was in place, we lack the data to estimate VO_2_ and further describe the effect of ΔT° on energy expenditure and gas exchange. However, given the significant effect of the ΔT° on heart rate and CO, this physiological response to mild cooling appears to be the most plausible explanation. Likewise, it would have been interesting to quantify the impact of ΔT° on microcirculatory parameters (such as mottles or capillary refilling time) to inform on the effect of ΔT° on skin perfusion assessment. Finally, we also acknowledge that UF_NET_ was not inserted as a covariate in regression and mediation models. Yet, UF_NET_ effect on HIRRT risk is mediated by decreased CO in preload dependent patients; hence its physiological impact and statistical association would, in our opinion, be accounted for in the presented models.

## Conclusions

In this single-center, prospective, observational study, setting the circuit temperature below body temperature (mild extracorporeal cooling) was significantly associated with a reduction in HIRRT risk during CRRT, and identified MAP and CI as plausible physiological pathways associated with this improvement. These results need to be confirmed in a prospective, multicenter, randomized controlled trial.

## Authors’ contributions

JCR, CG, LB and LF collected the data, interpreted the results and drafted the manuscript. JCR, LB and LF accessed and verified the underlying data. LB designed the study and performed the statistical analysis. TK, MC, GD, GC, MM, LC, HY and JCR interpreted the results, and revised the manuscript for important intellectual content. All authors had full access to all the data in the study and had final responsibility for the decision to submit for publication. All authors approved the final version to be published and agree to be accountable for all aspects of the work.

## Consent for publication

Not applicable.

## Ethical approval and consent to participate

The study was conducted in accordance with the Declaration of Helsinki and with local regulations. The study protocol was reviewed and approved by the Comité de Protection des Personnes Ile de France IV, ID-RCB 2017-A00483-50. All participants or their next-of-kin received all required information regarding study procedures and provided written informed consent to participate, including ancillary re-analysis of the original dataset. The study was registered at ClinicalTrials.gov, under the reference NCT03139123.

## Funding

None.

## Availability of supporting data

Source datasets are not publicly available due to ethical reasons. Further enquiries can be directed to the corresponding author at laurent.bitker@chu-lyon.fr. The authors vouch for the accuracy and completeness of the data.

## Declaration of competing interest

The authors declare no competing interest in relation with the work.

## Footnotes

Appendix A Supplementary data associated with this article can be found, in the online version, at http://dx.doi.org/10.1016/j.aicoj.2026.100108.
