# Supplementary material for: Extracorporeal circuit temperature gradient and hemodynamic instability during continuous renal replacement therapy: an observational study
Source: Ann Intensive Care. 2026 Jul 1;16:100108. doi: 10.1016/j.aicoj.2026.100108 (PMC13380051; doi:10.1016/j.aicoj.2026.100108)
Supplement: Supplementary file 1 [file mmc1.pdf]

Extracorporeal circuit temperature gradient and hemodynamic instability during continuous renal replacement therapy: an observational study.

Authors: Lorna Fraire, Téo Keraron, Matthieu Chivot, Hodane Yonis, Guillaume Chazot, Mehdi Mezidi, Louis Chauvelot, Guillaume Deniel, Jean-Christophe Richard, Laurent Bitker

SUPPLEMENTAL APPENDIX

Supplemental Methods 1. CRRT devices, fluids and settings

CRRT monitors (with integrated heating systems) were the Fresenius Multifiltrate Pro with an AV-1000S membrane (Fresenius Medical Care, Bad Homburg, Germany). Replacement fluids were Hemosol B0 (Baxter, Deerfield, IL, USA), or MultiBic K0 (Fresenius Medical Care, Bad Homburg Germany) for CVVH, and Ci-Ca Dialysate K2 (Fresenius Medical Care, Bad Homburg Germany) for CVVHD. CRRT effluent flow rate was set at 20-25 ml.kg-1.h-1, using ICU admission body weight. Blood flow rate was set at 200-300 ml.min-1 with CVVH, and at one twentieth (in ml.min-1) of the dialysate flow rate (in ml.h-1) for CVVHD with citrate regional anticoagulation, as per manufacturer recommendations.

Abbreviations: CRRT: continuous renal replacement therapy; CVVH: continuous veno-venous hemofiltration; CVVHD: continuous veno-venous hemodialysis.

Supplemental Methods 2. Mediation analysis methods

The DAG shows the *pre-treatment* confounders that were accounted for by the IPTW-based CBPS, and the *post-treatment* confounders associated with HIRRT risk (non-cardiovascular Sepsis-related Organ Failure Assessment [SOFA], arterial lactate concentration and norepinephrine dose). Importantly, fluid removal by UF_{NET} was not inserted in mediation models because its impact on HIRRT risk would also be theoretically mediated by cardiac output and preload dependence (1, 2).

Then, a multiple causal mediator analysis was performed on the longitudinal dataset, using the methodology developed by Imai and Yamamoto and mixed effects regression models, allowing the conjunct assessment of multiple mediators (i.e. cardiac output and MAP) (3). Temperature gradient dichotomized as being > 0°C or ≤ 0°C was used in the mediation models to predict HIRRT risk in the following 4 hours. This dichotomization (different from the 3 classes defined above) was justified by the fact that 1/ the HIRRT risk appeared lowest with this setting, and 2/ mediation analysis did not allow for the use of more than 2 classes of treatment (binary dependent variable). From these mediation models, the average direct (ADE) and average causal mediated (ACME) effects (on the probability scale) were quantified using simulations. Of note, because the outcome (HIRRT) was binary, mediated effects may differ slightly depending on treatment status (temperature gradient) due to the nonlinearity of the logistic model; we therefore reported the averaged mediated effect. Also, because we could not eliminate an interaction effect between temperature gradient and the mediator, we inserted an interaction of treatment × mediator in the models, and reported in the main analysis the resulting contrasts (i.e. how HIRRT risk would change if the mediator changed as it would with a ΔT° ≤ 0°C in a condition with ΔT° > 0°C.). Sensitivity analyses were performed in the subset of observations with normothermia.

Robustness of mediation models to unidentified confounders (i.e. sequential ignorability) were evaluated by estimating a correlation ρ parameter existing between the residuals of the mediator and outcome regressions (4).

Abbreviations: ΔT°: CRRT-to-core temperature gradient; CBPS: covariate balancing propensity score; DAG: direct acyclic graph; HIRRT: hemodynamic instability associated with renal replacement therapy; IPTW: inverse probability of treatment weighting; MAP: mean arterial pressure

Supplemental Table 1. Temperature and hemodynamics at baseline and during follow-up

**Variables**	**All patients**	**Predominant heat gain (ΔT° > 2°C)**	**Predominant neutral (ΔT° 0°–2°C)**	**Predominant heat loss (ΔT° ≤ 0°C)**
	N=42	N=16	N=12	N=14
Hemodynamics at baseline				
Mean arterial pressure, mmHg	70 [62–75]	71 [65–75]	68 [64–76]	70 [57–75]
Systolic arterial pressure, mmHg	110 [97–124]	114 [103–121]	106 [102–134]	106 [80–122]
Diastolic arterial pressure, mmHg	52 [45–58]	50 [44–56]	56 [48–61]	52 [46–56]
Cardiac index, $L.min^{-1}.m^{-2}$	2.8 [2.1–3.3]	2.7 [2.4–3.3]	2.8 [2.1–3.4]	2.8 [2.1–3.2]
Heart rate, min^{-1}	96 [74–113]	82 [72–97]	95 [72–109]	107 [96–119]
Stroke volume index, $ml.m^{-2}$	29 [24–38]	32 [25–45]	26 [22–41]	27 [20–33]
Preload dependent status, N (%)	22 (52%)	9 (56%)	4 (33%)	9 (64%)
Central venous pressure, mmHg	8 [6–10]	10 [5–10]	8 [7–9]	8 [6–10]
Extravascular lung water index, $ml.kg^{-1}$	11 [8.1–13.7]	10.4 [9–13.7]	13.3 [10.1–14.8]	11.5 [7.5–13]
Pulmonary vascular permeability index	2.2 [1.9–3]	2.2 [1.7–2.6]	2.4 [1.9–2.8]	2.4 [1.9–3.3]
Global end-diastolic volume index, $ml.m^{-2}$	664 [593–843]	765 [683–932]	732 [567–950]	597 [538–622]
Norepinephrine administration, N (%)	40 (95%)	15 (94%)	11 (92%)	14 (100%)
Norepinephrine dose (tartrate formula), $\mu g.kg^{-1}.min^{-1}$	0.54 [0.21–1.41]	0.56 [0.15–1.09]	0.3 [0.2–1.12]	0.76 [0.34–2.62]
Arterial lactate, $mmol.L^{-1}$	2.9 [1.5–5]	3 [1.9–4.7]	1.9 [1.4–3.3]	3.4 [1.6–6.8]
Temperature at baseline				
Core temperature at inclusion, °C	36.5 [35.8–36.9]	35.8 [35.6–36.3]	36.7 [36.0–36.8]	37.0 [36.3–37.4]
Core temperature < 36°C, N (%)	15 (36%)	9 (56%)	3 (25%)	3 (21%)
Core temperature 36–37.5°C, N (%)	24 (57%)	7 (44%)	9 (75%)	8 (57%)
Core temperature > 37.5°C, N (%)	3 (7%)	0 (0%)	0 (0%)	3 (21%)
Temperature gradient at inclusion, °C	1.3 [0.5–2.9]	2.9 [1.2–3.3]	1.3 [1.1–2.0]	0.6 [-0.4–2.0]
Temperature gradient > 2°C, N (%)	15 (36%)	9 (56%)	2 (17%)	4 (29%)
Temperature gradient 0–2°C, N (%)	20 (48%)	7 (44%)	10 (83%)	3 (21%)
Temperature gradient ≤0°C, N (%)	7 (17%)	0 (0%)	0 (0%)	7 (50%)
Temperature during follow-up				
Duration of follow-up per patient, h	119 [57–143]	140 [23–145]	69 [43–108]	131 [79–144]
Proportion of time spent in each temperature gradient category per patient, %*				
Temperature gradient > 2°C	34 [3–68]	77 [61–94]	9 [0–15]	8 [0–22]
Temperature gradient 0–2°C	35 [14–67]	16 [6–34]	84 [72–91]	18 [14–40]
Temperature gradient ≤0°C	6 [0–48]	0 [0–0]	2 [0–13]	57 [50–80]
HIRRT episodes during follow-up				
Total number of HIRRT episodes	214	92	54	68
Number of HIRRT episodes per patient	4 [3–8]	6 [3–10]	4 [3–4]	6 [3–8]

Data is median [interquartile range] or count (percentage).
Longitudinal groups of predominant temperature gradient categories (columns 2 to 4) were defined as fraction of time spent >2°C or ≤0°C greater than 30% of total follow-up. Groups were defined solely for descriptive purposes based on the predominant temperature gradient observed during follow-up and are not intended for direct comparison.
Norepinephrine doses are expressed in tartrate salt formula; divide the value by 2 to obtain the dose in base formula.

*: percentages may not sum to 100% because medians and interquartile ranges were computed separately within each temperature gradient category; however, percentages do sum to 100% within each patient.
ΔT°: temperature gradient; HIRRT: hemodynamic instability related to RRT

Supplemental Table 2. Association of hemodynamics and suspected confounders with HIRRT risk in the following 4 hours (univariate analysis)

**Variables (4-hourly observations)**	**HIRRT risk in the following 4h**	
	**Odd ratio [95% c.i.]**	**P value**
Mean arterial pressure, per 10 mmHg increase*		<0.01
First degree	0.09 [0.02–0.43]	
Second degree	1.01 [1.00–1.02]	
Cardiac index, per 0.1 $L.min^{-1}.m^{-2}$ increase	0.93 [0.91–0.96]	<0.01
Heart rate, per 10 min^{-1} increase*		<0.01
First degree	0.18 [0.09–0.35]	
Second degree	1.01 [1.01–1.01]	
Stroke volume index, per 10 $ml.m^{-2}$ increase	0.68 [0.54–0.86]	<0.01
Relative change in CCI during postural maneuver, per 1% increase	1.02 [1.01–1.04]	0.01
Preload dependent status (reference is preload independence)[‖]	1.60 [1.11–2.29]	0.01
Norepinephrine dose (tartrate), per 0.1 $\mu g.kg^{-1}.min^{-1}$ increase[§]	1.00 [0.98–1.02]	0.50
Temperature gradient, per 0.1°C increase	1.02 [1.01–1.03]	<0.01
Weighted temperature gradient, per 0.1°C increase	1.03 [1.01–1.05]	<0.01
**Relevant covariates**		
Age, per 1 year increase	1.01 [0.99–1.03]	0.30
Sepsis (reference is no sepsis) at time of inclusion	0.78 [0.46–1.33]	0.37
Invasive mechanical ventilation (reference is no invasive mechanical ventilation), collected 4-hourly	0.74 [0.39–1.41]	0.37
RASS score ≤ -4 (reference is > -4), collected 4-hourly	0.70 [0.41–1.22]	0.23
Core temperature, per 1°C increase, collected 4-hourly	0.96 [0.04–0.99]	0.01
CRRT modality is CVVHD (CVVH is the reference), collected 4-hourly	0.58 [0.31–1.11]	0.09
Daily non-cardiovascular SOFA score, per 1 point increase	0.94 [0.89–0.98]	0.01
Delay since last HIRRT < 8h (reference is ≥ 8h), collected 4-hourly	1.55 [1.07–2.24]	0.02
Daily lactate, per 1 $mmol.L^{-1}$ increase	2.27 [1.09–4.72]	0.04

*: quadratic factor in the model
‖: defined as a CCI increase > 10% during the postural maneuver
§: Norepinephrine doses are expressed in tartrate salt formula; divide the value by 2 to obtain the dose in base formula.
Mixed effects generalized linear regression models were run on 10 imputed datasets (N=970 observations in each dataset, due to the absence of HIRRT follow-up in the next 4h following the last time point of follow-up), with the variable of interest as the fixed effect, HIRRT as the dependent variable, visit number as the random slope nested in a random intercept corresponding to the patient identification number. Variables were scaled and centered prior to regression (norepinephrine required additional transformation using the Box-Cox method due to leftward skewness). Models' goodness-of-fit were checked using Hartig et al. method (R package *DHARMa*). Fixed effects were then pooled using Rubin's rule and descaled to the original hemodynamic parameter scale. Weighting of temperature gradient was performed using the CBPS method to adjust for the effects of pre-treatment confounders on temperature gradient. P values were bootstrapped over 500 replicate datasets.
95% c.i.: 95% confidence interval; CCI: continuous cardiac index by pulse contour analysis; HIRRT: hemodynamic instability related to renal replacement therapy; RASS: Richmond's analgesia and sedation scale; SOFA= sepsis-related organ failure assessment.

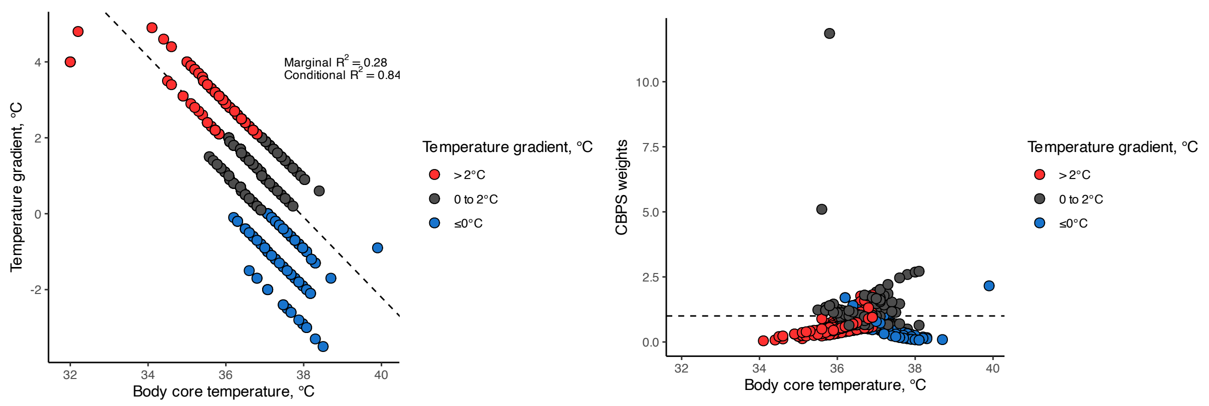

Supplemental Figure 1. Correlation between core temperature and temperature gradient, and estimated CBPS weights applied to temperature gradient as a function of pre-treatment cofounders

The figure shows the correlation (dotted line) between core temperatures and temperature gradients in panel A, and the weight values applied to temperature gradient (in panel B) in models to correct for the confounding effect of pre-treatment cofounders (core temperature, age, sepsis, RASS, invasive mechanical ventilation, CRRT modality), with the horizontal dotted line representing a CBPS weight of 1. Data points are also categorized based on the temperature gradient category (>2°C in red, between 0°C and 2°C in grey, and ≤0°C in blue). Weights were determined using the covariate balancing propensity score methods for continuous treatments (Fong et al., 2018), and applied using inverse probability of treatment probability (IPTW). For instance, the figure shows that in observations with hyperthermia (> 37.5°C), higher weights were adjudged to observations with a gradient above 0°C. CRRT: continuous renal replacement therapy;
CBPS: covariate balancing propensity score; RASS: Richmond analgesia and sedation scale.

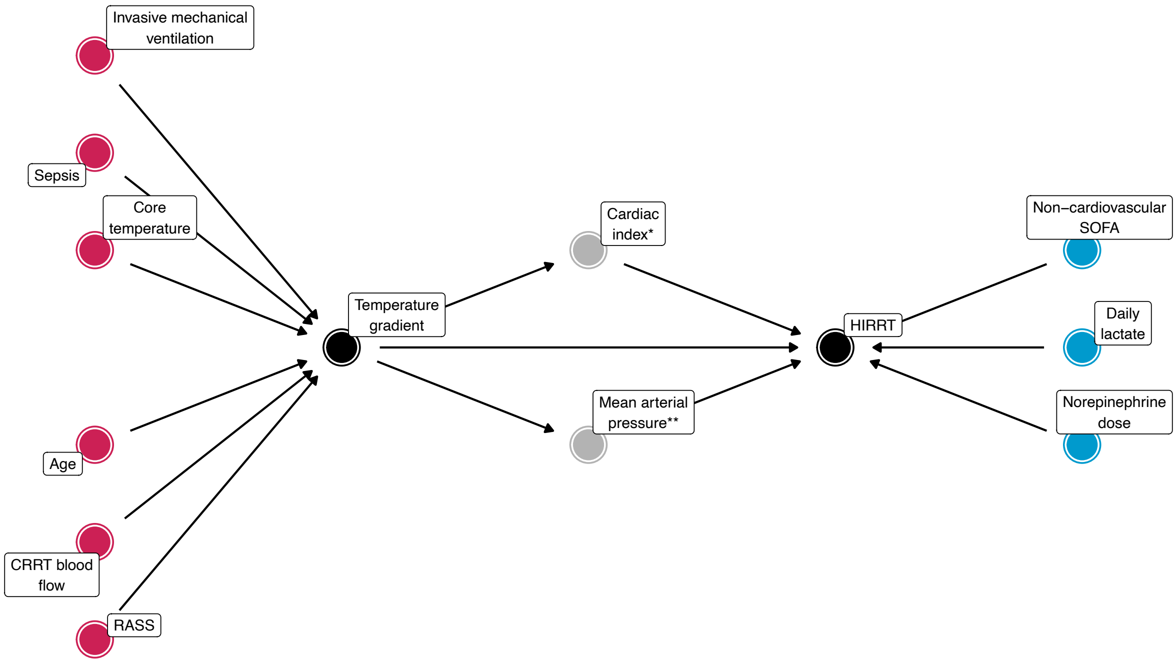

Supplemental Figure 2. Directed acyclic graph

The figure shows the hypothesized causal relation between temperature gradient set on the CRRT monitor and the HIRRT risk, mediated through mean arterial pressure and cardiac index. Given the fact that mean arterial pressure and cardiac index are mathematically related by the equation of movement, a sensitivity analysis using diastolic arterial pressure (as a surrogate of systemic vascular resistances) instead of mean arterial pressure was performed. In mediation models (* and **), both mediators alternatively acted as the main mediator, while the other acted as the alternate mediator. Core temperature and other covariates acted as a potential pre-treatment confounder (in red), while norepinephrine dose, non-cardiovascular SOFA score and arterial lactate concentration acted as post-treatment confounders, interacting with both the outcome and mediators (in blue). Interactions between mediators and treatment were also accounted for.

SOFA: sepsis-related organ failure assessment

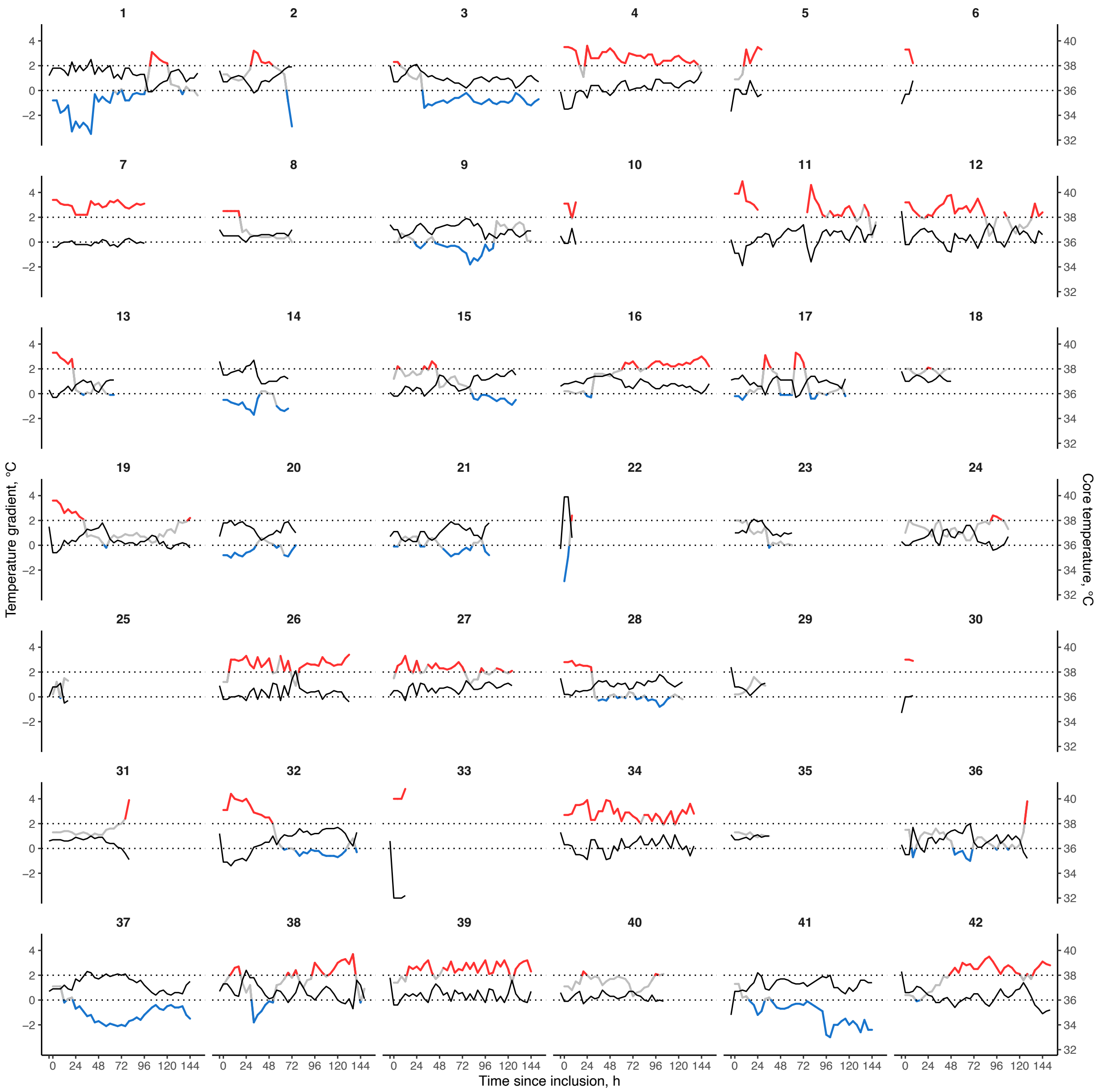

Supplemental Figure 3. Individual course of core temperature and temperature gradient in all patients

The figure shows the individual values of 4-hourly core temperature (black line, scale on the right-hand side *x* axis) and temperature gradient (blue, red and grey lines, scale on the left-hand side *y* axis) in all enrolled patients (N=42). The dotted horizontal lines correspond to the gradient cutoffs of 0°C and 2 °C. Gradients > 2°C are represented in red, those ≤ 0°C in blue.

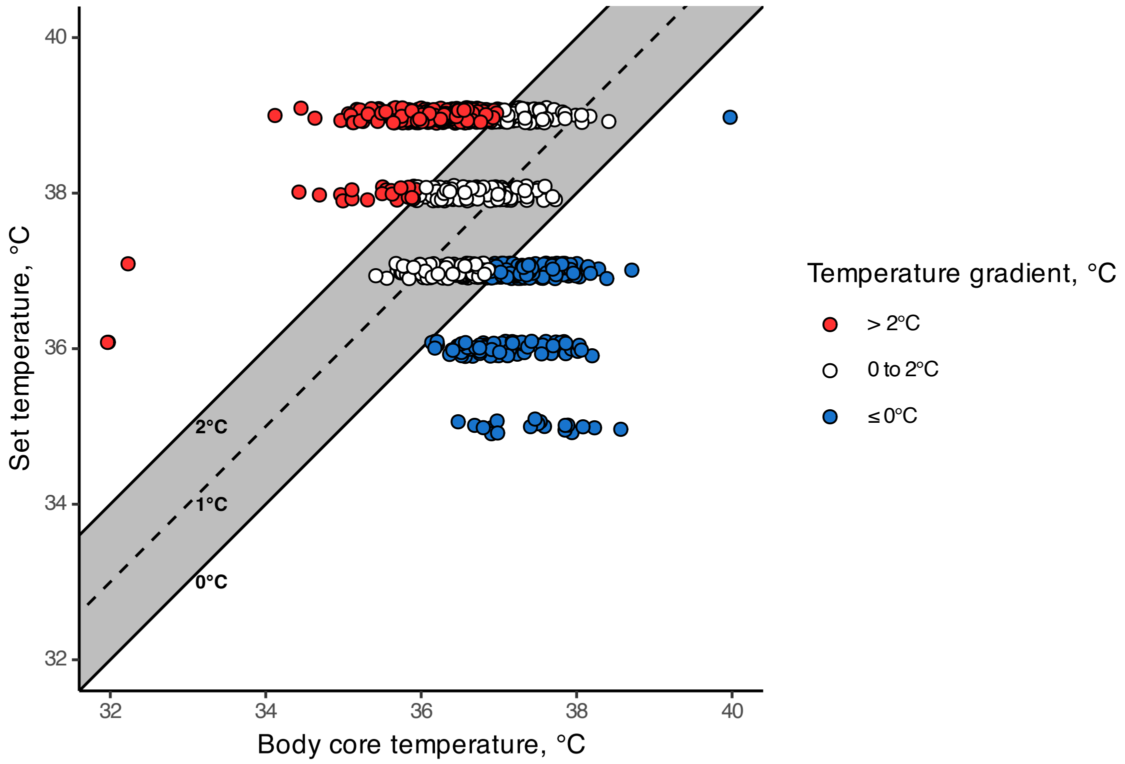

Supplemental Figure 4. Core temperature, CRRT set temperature and temperature gradient

The figure shows the relationship between body core temperature (measured on the PiCCO® device in arterial femoral bloodstream) and the set temperature on the CRRT monitor. A positive temperature gradient indicates that the core temperature was below the set temperature on the CRRT monitor. The grey shade corresponds to temperature gradient between 0°C and 2°C. Data points are categorized based on the temperature gradient value (>2°C in red, between 0 and 2°C in white, and ≤0°C in blue). Of note, no negative gradient was observed when core temperature was below 36°C, and gradients >2°C were not observed when core temperature was >37.5°C. This is related to the range of circuit temperature settings on the CRRT device, restricted between 35°C and 39°C.

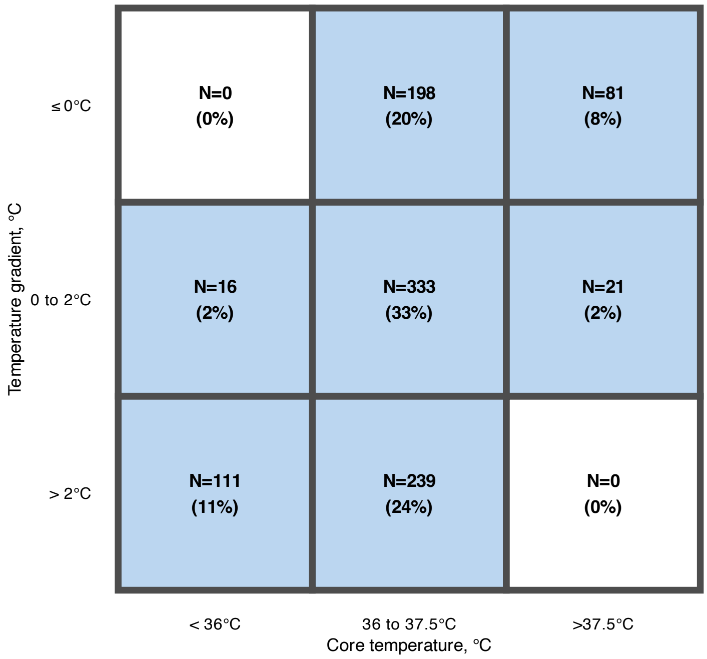

Supplemental Figure 5. Distribution of core temperature and temperature gradient categories.
The figure shows the number and percentage of observations in each of the 3 gradient categories (≤ 0°C, 0 to 2 °C and > 2°C) and the 3 core temperature categories (<36°C, 36°C to 37°C, and > 37.5°C) in the longitudinal data set (N=1012 observations, 13 missing values [1%]). The percentages are reported to the total number of observations.

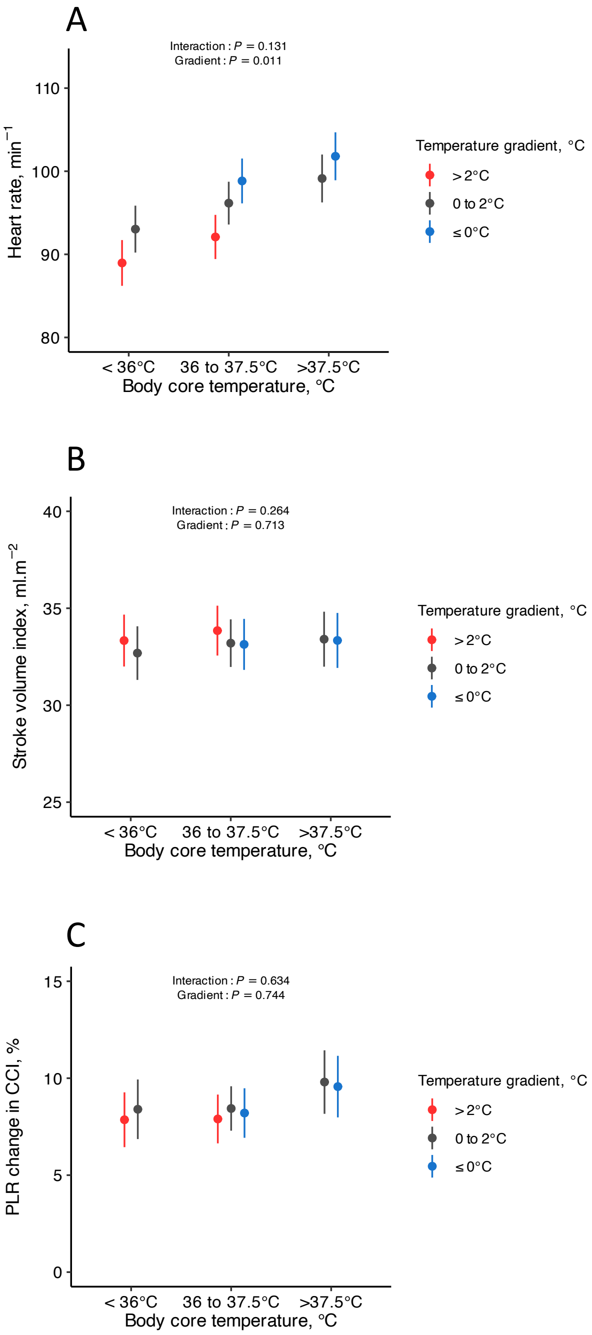

Supplemental Figure 6. Association of hemodynamic parameters with temperature gradient and core temperature.

The figure shows the mean value of heart rate (A), stroke volume index (B) and relative change in continuous cardiac index during a postural maneuver (C) based on the core temperature category (x axis) and the temperature gradient (>2°C in red, between 0 and 2°C in grey, and ≤0°C in blue) during longitudinal follow-up (4-hourly observations). The represented values are the marginal means (and associated standard error) determined from a mixed effect model with the hemodynamic parameter as the dependent variable, temperature gradient category and core temperature category as the explanatory variables (with an interaction term if significant). Models' random effects were a random slope of visit number nested in a random intercept corresponding to the patient identification number. An offset was inserted in the model, corresponding to the hemodynamic parameter value at baseline. Marginal means were determined in 10 imputed datasets (N=1012 observations in each dataset), and pooled using Rubin's method. Variables were scaled prior to regression (with additional Box-Cox transformation for norepinephrine due to leftward skewness) and descaled after. For all variables, interaction between the 2 explanatory variables was checked. P values were bootstrapped over 500 replicate datasets. Collinearity between the 2 categorical variables was eliminated using a variance inflation factor < 3.

CCI: continuous cardiac index; PLR: passive leg raising test (postural maneuver)

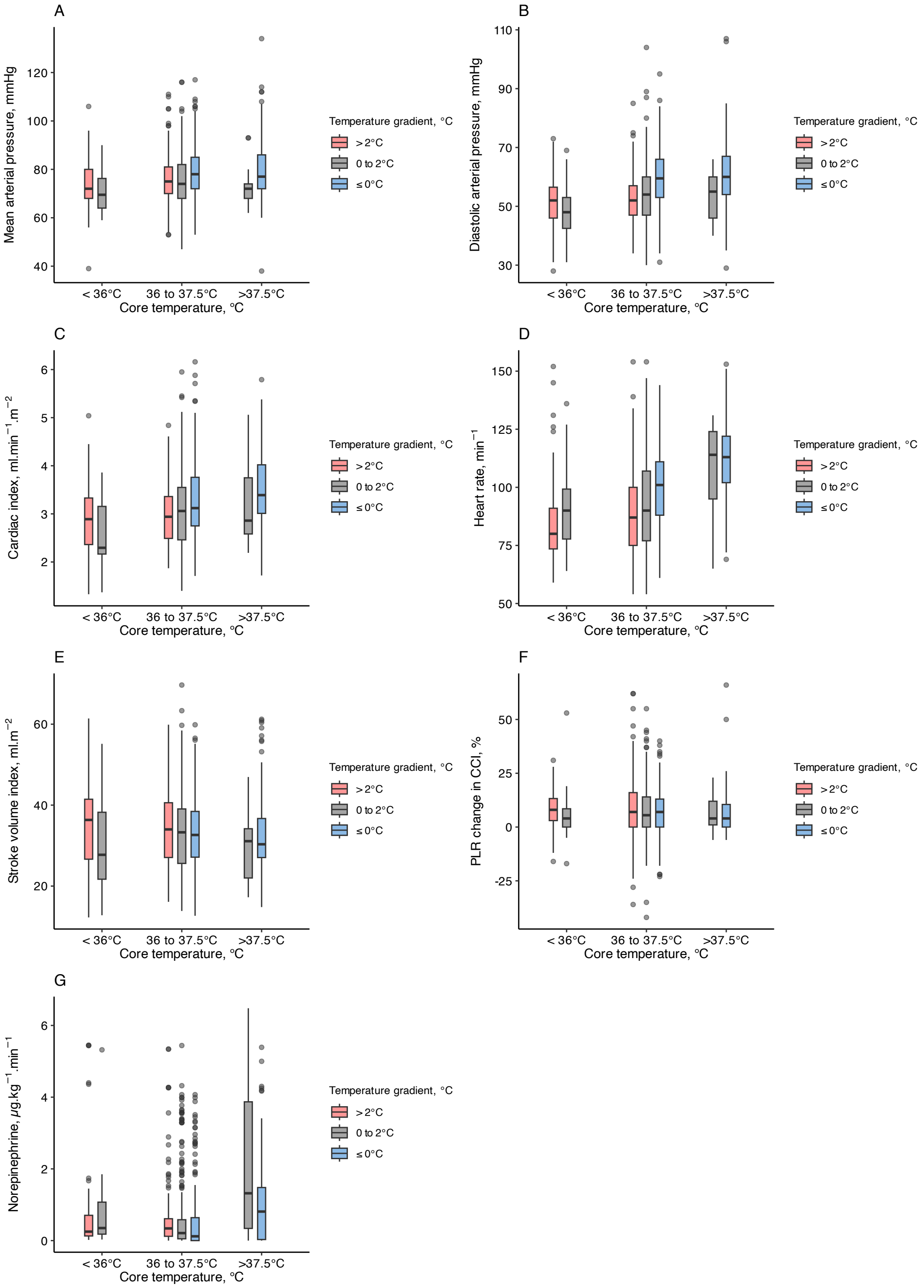

Supplemental Figure 7. Observed hemodynamic values based on the temperature gradient and core temperature categories

The figure shows the observed median value of mean arterial pressure (A), diastolic arterial pressure (B), cardiac index (C), heart rate (D), stroke volume index (E), relative change in CCI during a postural maneuver (F) and norepinephrine dose (G) as a function of the core temperature category (x axis) and the temperature gradient (> 2°C in red, between 0 and 2°C in grey, and ≤ 0°C in blue) during longitudinal follow-up (4-hourly observations, N=1012). Norepinephrine doses are expressed in tartrate salt formula; divide the value by 2 to obtain the dose in base formula.

CCI: continuous cardiac index by pulse contour analysis; PLR: passive leg raising test (postural maneuver)

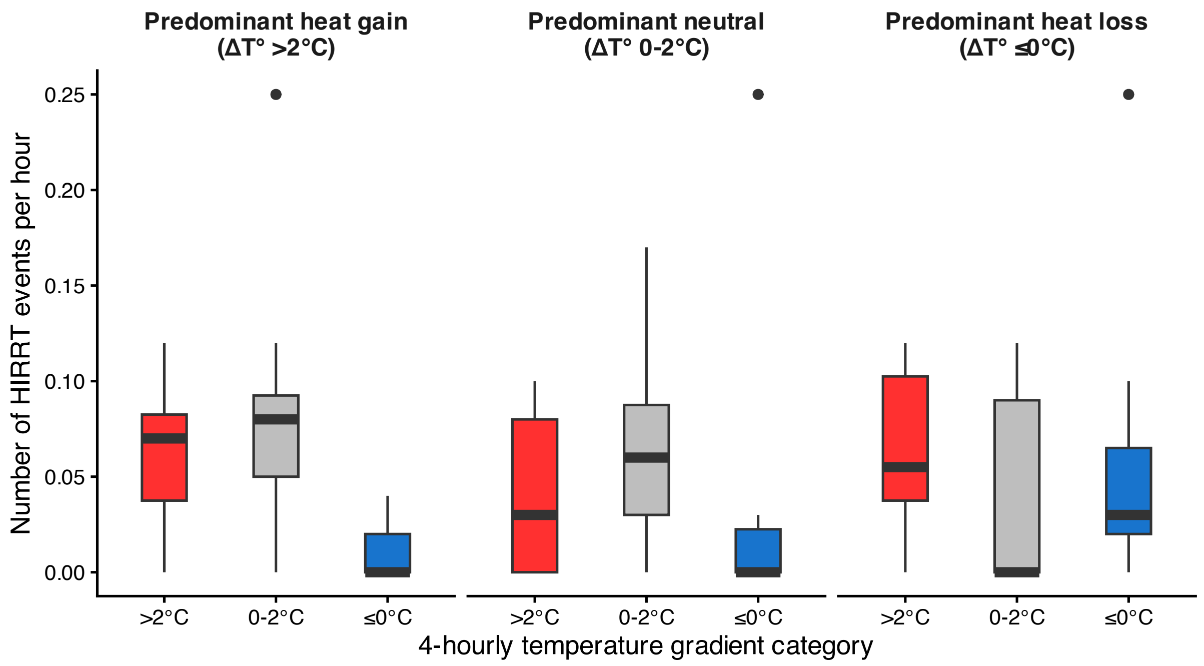

Supplemental figure 8. Rate of HIRRT events by 4-hourly temperature gradient category, in each predominant temperature gradient group

The figure shows the rate of HIRRT events (normalized to the number spent in each 4-hourly ΔT° category) in each of the 3 predominant ΔT° groups (predominantly heat gain, neutral or heat loss). The total number of HIRRT events was 214 (with a mean rate of 0.05 events per hour over the cumulative follow-up duration). 4-hourly ΔT° category were > 2°C in red, between 0°C and 2°C in grey, and ≤ 0°C in blue. Longitudinal groups of predominant temperature gradient categories (columns 2 to 4) were defined as fraction of time spent >2°C or ≤0°C greater than 30% of total follow-up. Groups were defined solely for descriptive purposes based on the predominant temperature gradient observed during follow-up and are not intended for direct comparison.

ΔT°: temperature gradient; HIRRT: hemodynamic instability related to RRT

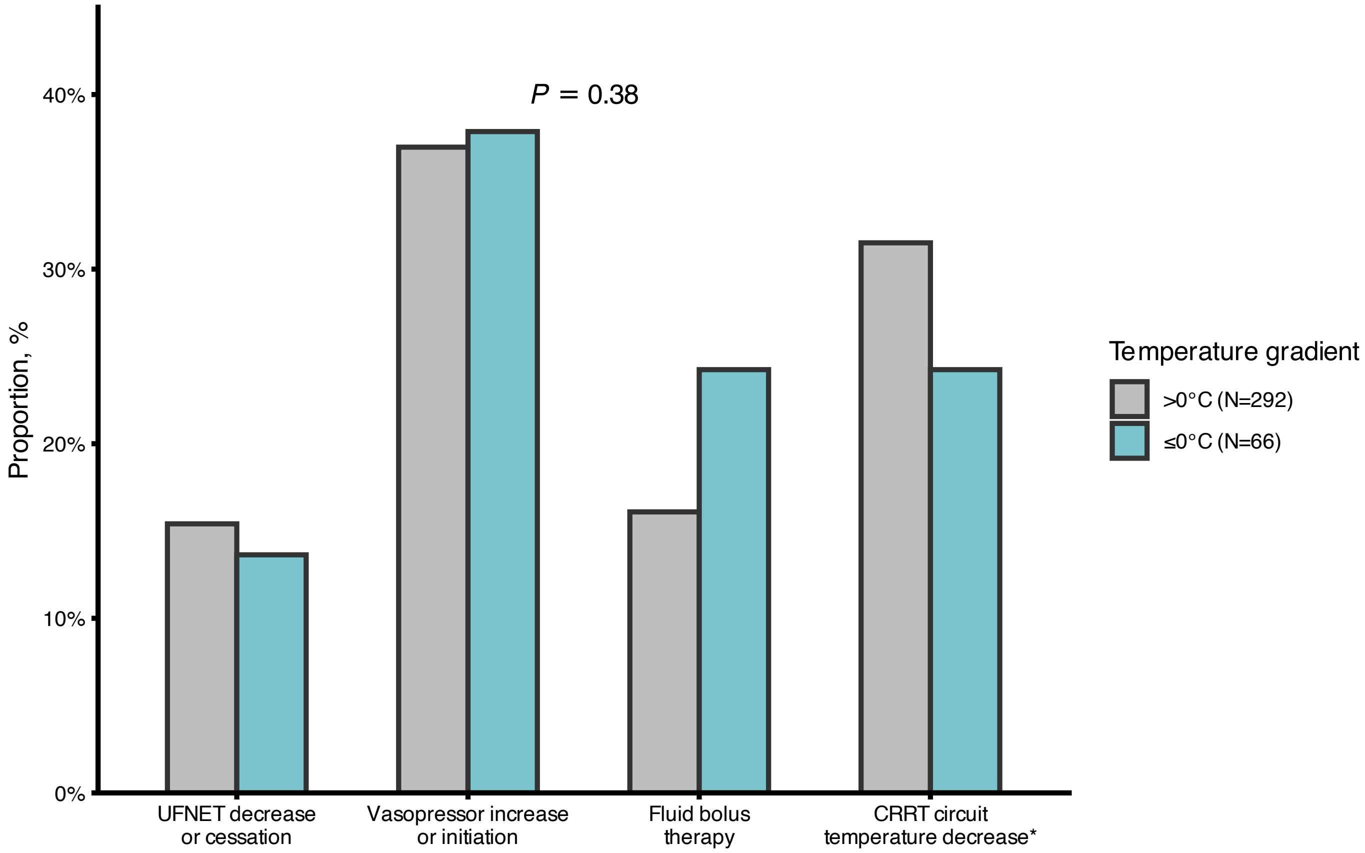

Supplemental Figure 9. Distribution of therapeutic interventions performed to treat HIRRT episodes.
The figure shows the proportion of each intervention used to define HIRRT (alongside a MAP <65 mmHg): UF_{NET} decrease or cessation, vasopressor initiation or dose increase, and fluid bolus administration. A fourth category (CRRT circuit temperature decrease) was added for descriptive purposes, to illustrate how the occurrence of a HIRRT episode may have influenced the CRRT temperature setting applied in the subsequent 4-hour period. Grey bars correspond to HIRRT episodes occurring during observation periods with a temperature gradient >0°C; blue bars to those with a temperature gradient ≤0°C. Proportions are reported relative to the total number of HIRRT episodes in each group and may exceed 100%, as multiple interventions could be performed simultaneously by the treating team. The P value evaluates the overall difference in distribution of therapeutic interventions between the 2 temperature gradient groups.
*CRRT circuit temperature decrease was defined as a reduction in the CRRT temperature setting between the observation period of interest and the immediately following study time point.
UF_{NET}: net ultrafiltration.

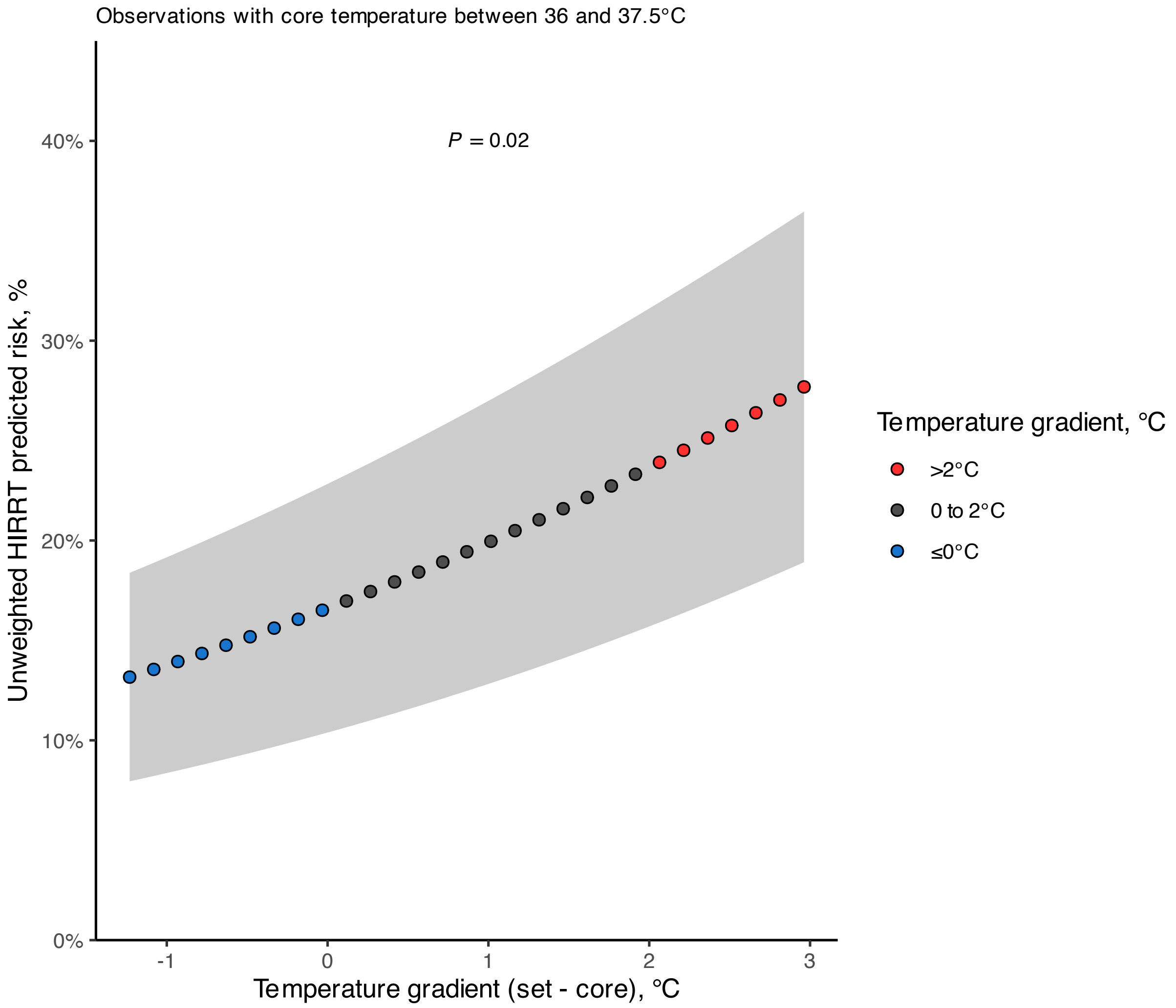

Supplemental Figure 10. Association of temperature gradient with longitudinal HIRRT risk during follow-up (unweighted analysis in observations with normal core temperature)

The figure shows the predicted risk of HIRRT associated with temperature gradient during follow-up in observations with a normal core temperature. Gradient categories are also represented. The grey shade represents the standard deviation of the prediction. Prediction was performed using a generalized linear regression mixed effects model, with HIRRT as the dependent variable, temperature gradient as the explanatory variable, and applied to 10 imputed datasets. Models' random effects were a random slope of visit number nested in a random intercept corresponding to the patient identification number. Model coefficients were not weighted with CBPS. Model coefficients were then pooled and applied to a synthetic dataset with temperature gradient varying between the lowest and highest value observed in the cohort. For each imputed datasets, the C-statistics (and its 95% confidence interval, Delong's method) and the Kolmogorov–Smirnov goodness-of-fit test were performed, and their results pooled. Final model's C-statistics: 0.56 [95% confidence interval: 0.53–0.59], Kolmogorov–Smirnov test: *P*=0.41.

CBPS: covariate balancing propensity score; HIRRT: hemodynamic instability associated with renal replacement therapy

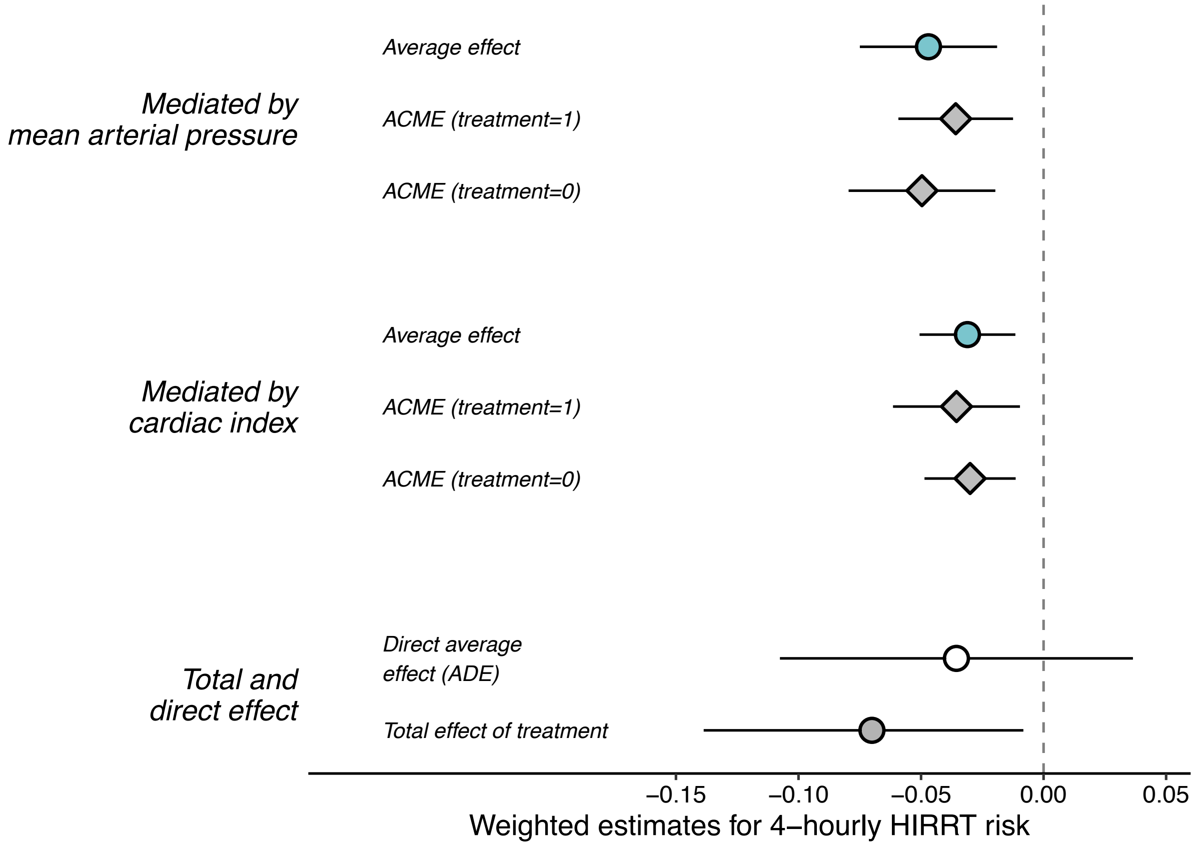

Supplemental Figure 11. Exploratory mediation analysis

The figure shows the weighted estimates (ACME, average causal mediated effect) associated with a temperature gradient ≤0° (blue dots, reference is > 0°C), mediated by mean arterial pressure (MAP, upper panel) and cardiac index (CI, middle panel). The estimate corresponds to the absolute risk variation (*x* axis) associated with a 1 standard deviation increase in MAP (+10 mmHg) and CI (+0.75 L.min^{-1}.m^{-2}), respectively. The figure also shows the average direct (white dot) and total effect (grey dot) of a ΔT° ≤ 0°C on HIRRT risk. The vertical dotted line identifies the absence of effect. The treatment specific mediated effects (grey diamonds) were reported given the fact that the model's outcome was binary and that a interaction term of *temperature gradient × mediator* was used; it shows how the effect of the mediator (increasing MAP or CI) on HIRRT risk would be modified if ΔT° was ≤ or > 0°C (treatment = 1 and 0, respectively). The mediation model was designed using the methodology developed by Imai and Yamamoto (2013), which allows the incorporation of alternative mediators when performing multiple causal analysis. Hence, 2 models were designed: one with the mean arterial pressure as the main mediator, and cardiac index as the alternative mediator. The second model did the opposite (cardiac index as the main mediator and mean arterial pressure as the alternative mediator). An interaction term between the treatment (temperature gradient) and the mediator (mean arterial pressure or cardiac index) was also included. The mediation model incorporated the following post-treatment confounders: non cardiovascular SOFA, norepinephrine dose, and daily arterial lactate concentration. The models were also weighted for pre-treatment cofounders (weights determined using the CBPS method). Models were evaluated on 10 imputed datasets, and their results pooled using Rubin's rule. Nonparametric confidence intervals were bootstrapped over 600 replicate datasets. Sensitivity analyses showed that the models were potentially susceptible to potential unaccounted-for confounders ($R^{2}*$ and $\tilde{R}^{2}$ = 0.1).

ACME: average causal mediated effect; ADE: average direct effect; CI: cardiac index; HIRRT: hemodynamic instability related to renal replacement therapy; MAP: mean arterial pressure; SOFA: sepsis-related organ failure assessment

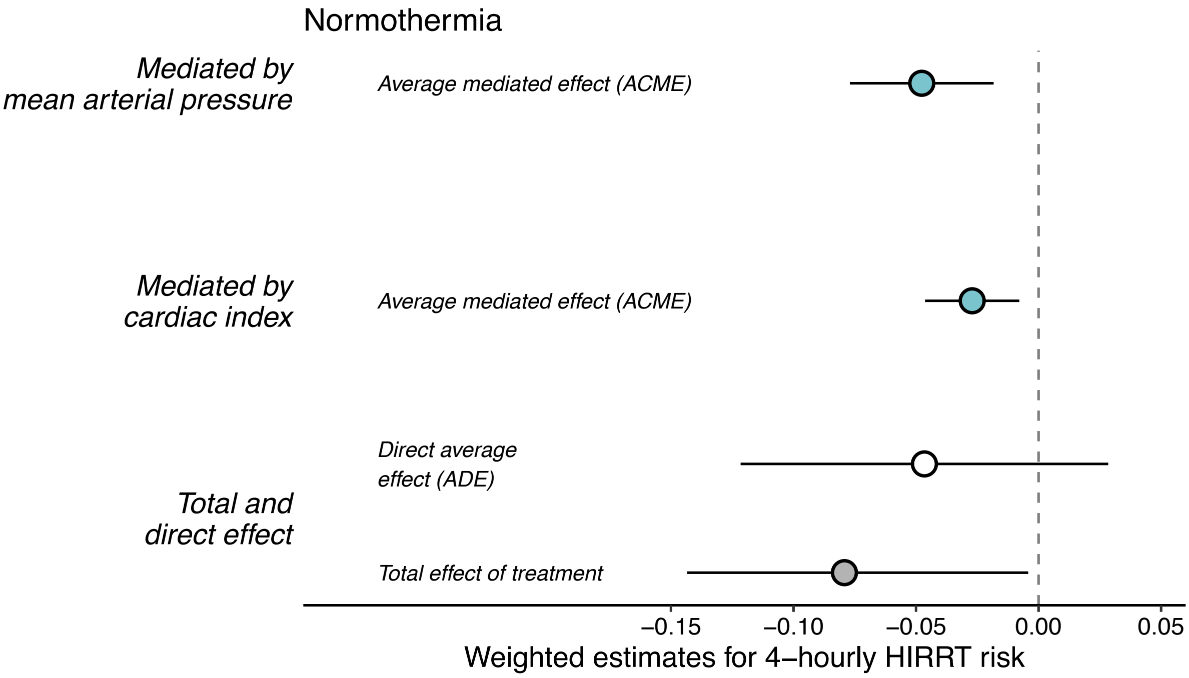

Supplemental Figure 12. Mediation analysis in 4-hourly observations with normothermia.
The figure shows the sensitivity analysis performed in the subset of observations with normothermia (core temperature between 36°C and 37.5°C). The methodology used follows exactly that presented in the main analysis, and its interpretation is the same.

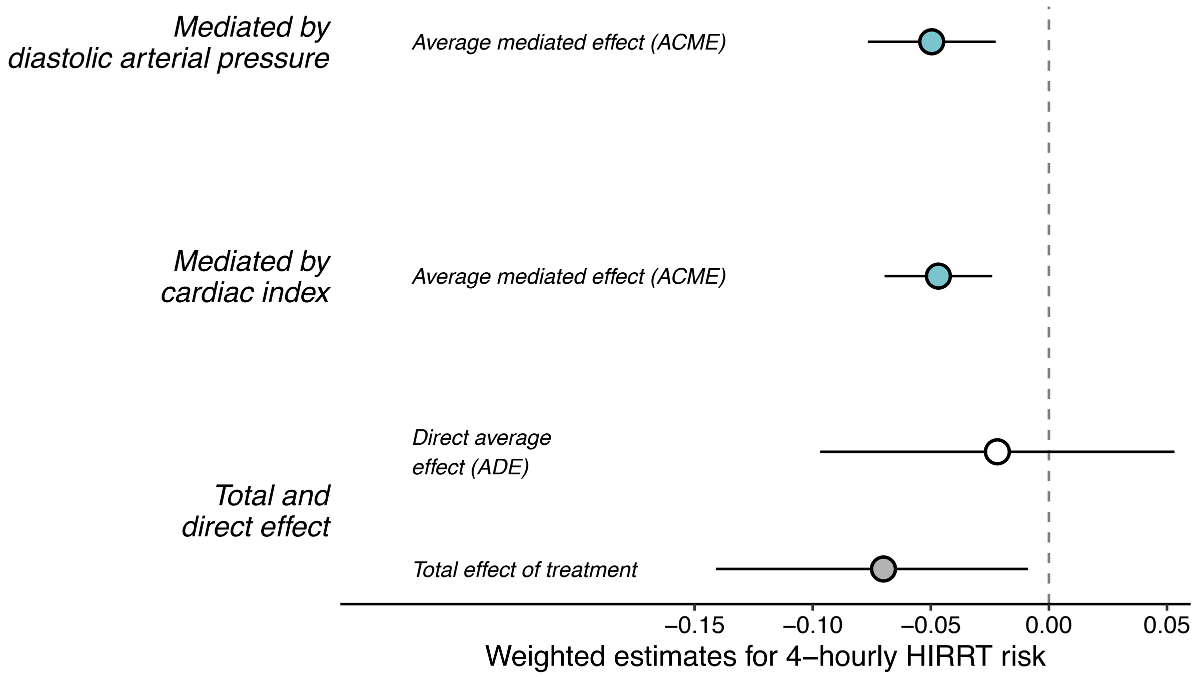

Supplemental Figure 13. Mediation analysis in 4-hourly observations using diastolic arterial pressure instead of mean arterial pressure.
The figure shows a sensitivity analysis performed using diastolic arterial pressure (as a proxy of systemic vascular2026 resistances) instead of mean arterial pressure. The methodology used follows exactly that presented in the main analysis, and its interpretation is the same.

Supplemental References

1. Chazot G, Bitker L, Mezidi M, Chebib N, Chabert P, Chauvelot L, et al. Prevalence and risk factors of hemodynamic instability associated with preload-dependence during continuous renal replacement therapy in a prospective observational cohort of critically ill patients. Ann Intensive Care. 2021;11:95.
2. Bitker L, Dupuis C, Pradat P, Deniel G, Klouche K, Mezidi M, et al. Fluid balance neutralization secured by hemodynamic monitoring versus protocolized standard of care in patients with acute circulatory failure requiring continuous renal replacement therapy: results of the GO NEUTRAL randomized controlled trial. Intensive Care Med. 2024;50:2061-72.
3. Imai K, Keele L, Tingley D. A general approach to causal mediation analysis. Psychol Methods. 2010;15:309-34.
4. Imai K, Yamamoto T. Identification and Sensitivity Analysis for Multiple Causal Mechanisms: Revisiting Evidence from Framing Experiments. Politi Anal. 2013;21:141-71.
